# Minimizing activation of overlying axons with epiretinal stimulation: The role of fiber orientation and electrode configuration

**DOI:** 10.1371/journal.pone.0193598

**Published:** 2018-03-01

**Authors:** Timothy B. Esler, Robert R. Kerr, Bahman Tahayori, David B. Grayden, Hamish Meffin, Anthony N. Burkitt

**Affiliations:** 1 Department of Biomedical Engineering, The University of Melbourne, Parkville, Victoria, Australia; 2 Seer Medical, Melbourne, Victoria, Australia; 3 Monash Institute of Medical Engineering, Monash University, Clayton, Victoria, Australia; 4 Centre for Neural Engineering, The University of Melbourne, Parkville, Victoria, Australia; 5 National Vision Research Institute, Australian College of Optometry, Carlton, Victoria, Australia; 6 ARC Centre of Excellence for Integrative Brain Function, Optometry & Vision Science, The University of Melbourne, Parkville, Victoria, Australia; Universidade Federal do ABC, BRAZIL

## Abstract

Currently, a challenge in electrical stimulation of the retina with a visual prosthesis (bionic eye) is to excite only the cells lying directly under the electrode in the ganglion cell layer, while avoiding excitation of axon bundles that pass over the surface of the retina in the nerve fiber layer. Stimulation of overlying axons results in irregular visual percepts, limiting perceptual efficacy. This research explores how differences in fiber orientation between the nerve fiber layer and ganglion cell layer leads to differences in the electrical activation of the axon initial segment and axons of passage. *Approach*. Axons of passage of retinal ganglion cells in the nerve fiber layer are characterized by a narrow distribution of fiber orientations, causing highly anisotropic spread of applied current. In contrast, proximal axons in the ganglion cell layer have a wider distribution of orientations. A four-layer computational model of epiretinal extracellular stimulation that captures the effect of neurite orientation in anisotropic tissue has been developed using a volume conductor model known as the cellular composite model. Simulations are conducted to investigate the interaction of neural tissue orientation, stimulating electrode configuration, and stimulation pulse duration and amplitude. *Main results*. Our model shows that simultaneous stimulation with multiple electrodes aligned with the nerve fiber layer can be used to achieve selective activation of axon initial segments rather than passing fibers. This result can be achieved while reducing required stimulus charge density and with only modest increases in the spread of activation in the ganglion cell layer, and is shown to extend to the general case of arbitrary electrode array positioning and arbitrary target volume. *Significance*. These results elucidate a strategy for more targeted stimulation of retinal ganglion cells with experimentally-relevant multi-electrode geometries and achievable stimulation requirements.

## Introduction

**Fig 1:**
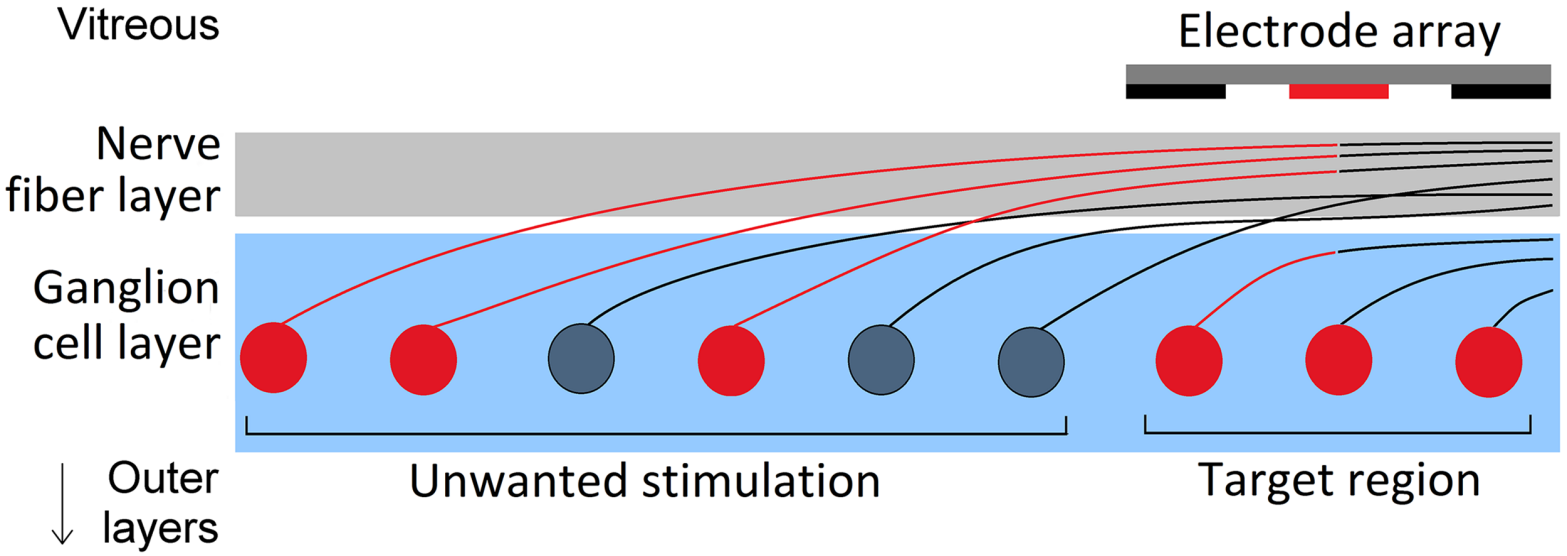
Unwanted stimulation of retinal ganglion cell axons of passage. Retinal ganglion cell somas and axon initial segments represent the target regions for epiretinal stimulation (region shaded blue). Activation of passing axons in the nerve fiber layer (gray shaded region) results in long, arc-shaped visual percepts and degradation of the quality of artificial vision. Retinal ganglion cell axon bundles in the nerve fiber layer that pass close to stimulating electrodes may be stimulated preferentially to target locations in the ganglion cell layer. Activated retinal ganglion cells are colored red. Simulations presented in this research use epiretinal multi-electrode arrays (100 *μ*m diameter, 200 *μ*m pitch). Note that the orientation of initial axonal segments is much more varied in reality than shown in this schematic.

There has been significant progress over the past decade in the development of retinal prostheses for those with retinal pathologies such as Retinitis Pigmentosa. Clinical trials of retinal prostheses have found that patients can reliably report visual percepts arising from stimulation and can perform simple identification tasks [1–7]. Although progress to date is highly encouraging, many aspects of the performance of retinal prostheses remain limited, hinging on the ability of these devices to target either specific retinal cell types [8, 9] or more precise retinal volumes [2, 4, 10, 11]. In the case of epiretinal stimulation, a factor limiting performance is the inability of electrical stimulation to preferentially activate target neuronal structures in the ganglion cell layer (GCL), such as the axon initial segment (AIS), while avoiding activation of overlying axons in the nerve fiber layer (NFL) [2, 10–19], illustrated in Fig 1.

Since the axons of retinal ganglion cells (RGCs) traverse the inner surface of the retina in the NFL, epiretinal electrical stimulation faces the challenge of stimulating the deeper, favorably-organized GCL while minimizing activation of axons of passage (AOPs) in the NFL. Recipients of epiretinal implants commonly describe irregular visual percept shapes due to stimulation of axons of passage [13, 15, 20, 21]. This effect has been confirmed experimentally and in simulations, and results in a reduction in the spatial selectivity of epiretinal stimulation [10, 11, 15–17, 20–22]. Recent experimental findings by Grosberg et al. [10] have both reaffirmed the existence of this problem while suggesting that it may be overcome using electrode stimulus amplitudes carefully tuned via detection of RGC activation in response to stimulation.

Owing to the presence of an experimentally-observed, high-density sodium channel band, the AIS has been shown to be the most excitable part of a RGC [23]. Hence, it is important to compare the activation of AOPs with the AIS, as opposed to the soma. A potential way to minimize activation of AOPs is to take advantage of differences in neurite orientation in the NFL and GCL. The direction of overlying axon tracts represents the dominant fiber orientation in a given location in the NFL. These axons are packed together as mostly parallel fibers [10, 11, 13]. As a result, current flow from epiretinal electrical stimulation spreads through retinal tissue in a highly anisotropic way. In contrast to the distal RGC AOPs in the NFL, proximal axon regions, such as the AIS located in the GCL, have a much wider distribution of orientations as they pass out from the soma. Based on these anisotropic tissue characteristics, it is expected that the orientation of a neurite in retinal tissue can have a significant effect on its activation. However, a common approximation employed by existing computational models of epiretinal stimulation is that the retinal layers are isotropic [11, 16, 18, 24]. In order to assess the effect of neurite orientation and its interaction with different multi-electrode configurations, computational models of current flow and axonal activation must be developed that can describe the anisotropic characteristics of key retinal layers.

In the absence of sufficient data to model the anisotropy of the NFL, an alternative approach is to derive layer anisotropy from first principles using a geometric description of the axonal units that comprise the tissue. The cellular composite model, introduced by Meffin et al. [25–28], provides a modeling framework that accomplishes this while addressing a number of limitations of conventional volume conductor models. Common two-stage approaches, such as those that combine a linear, numerically-integrated volume model for extracellular voltage (typically simulated in COMSOL Multiphysics) with a conductance-based neuron model (typically simulated in NEURON), are confounded by inconsistencies between the two simulation stages [28, 29]. This effect was exemplified by Tahayori et al. [28], who showed that, in the presence of model inconsistency, the choice of simulating either extracellular current or extracellular voltage could result in changes in simulated membrane potential by up to an order of magnitude. This is caused by a discrepancy between the tissue impedance underlying the two simulation stages. To more accurately capture the structural and temporal properties of neural tissue and to guarantee model self-consistency, the cellular composite model maps extracellular current to voltage using an expression for impedance derived directly from the geometry and physiology of the tissue’s microscopic constituent axons, providing consistent descriptions of both extracellular voltage/current and neural activation. Here, we present a multi-layered generalization of the cellular composite for which a closed-form solution exists in the Fourier domain. This solution yields modeling results that are more readily interpretable than three-dimensional finite-element model simulations. Furthermore, the relative computational efficiency of this approach allows for large-scale parameter sweeps. Although not explored in this research, the simulation approach presented here also lends itself to (closed-form) model inversion, which can be applied to studying the inverse stimulation problem: determining optimal stimulus currents given desired tissue activation patterns.

In addition to intrinsic tissue anisotropy, RGC activation will also depend on the orientation of the applied electric field. One existing modeling study by Rattay and Resatz [11] assessed the influence of electric field orientation with respect to neurites in the NFL. This study showed that, by orientating long, rectangular electrodes parallel to axons in the NFL, the activation of those axons could be reduced. The basis for this result is that the membrane potential response of an axon to extracellular stimulation is largely determined by the activating function: the second spatial derivative of the extracellular potential along the axon’s length [30]. By ‘flattening’ the extracellular potential along the length of the axon using long parallel electrodes, the activation of the axon is minimized.

The aim of this paper is to demonstrate a multi-electrode stimulation strategy for the avoidance of activation of axon bundles, while achieving focal activation of axon initial segments the in GCL. We present a model that captures both the effect of electric field orientation imposed by multi-electrode stimulation and the effect of the highly anisotropic geometry of the NFL. Increased sensitivity to stimulation at the AIS attributed to the high-density sodium channel band [23] has been incorporated into the model via an adjustment of threshold membrane potentials. Simulation results are presented that illustrate the achievable levels of preferential activation for one-, two-, and four-electrode configurations. An exploration of the effect of electrode-retina separation distance and pulse duration are presented, as well as the effect of different strategies on key performance metrics: required stimulus charge, GCL activation, and activation radius. The proposed multi-electrode array strategy is then validated against a more general set of electrode geometries and target volumes.

## Methods

### Distribution of orientations in the ganglion cell layer

**Fig 2:**
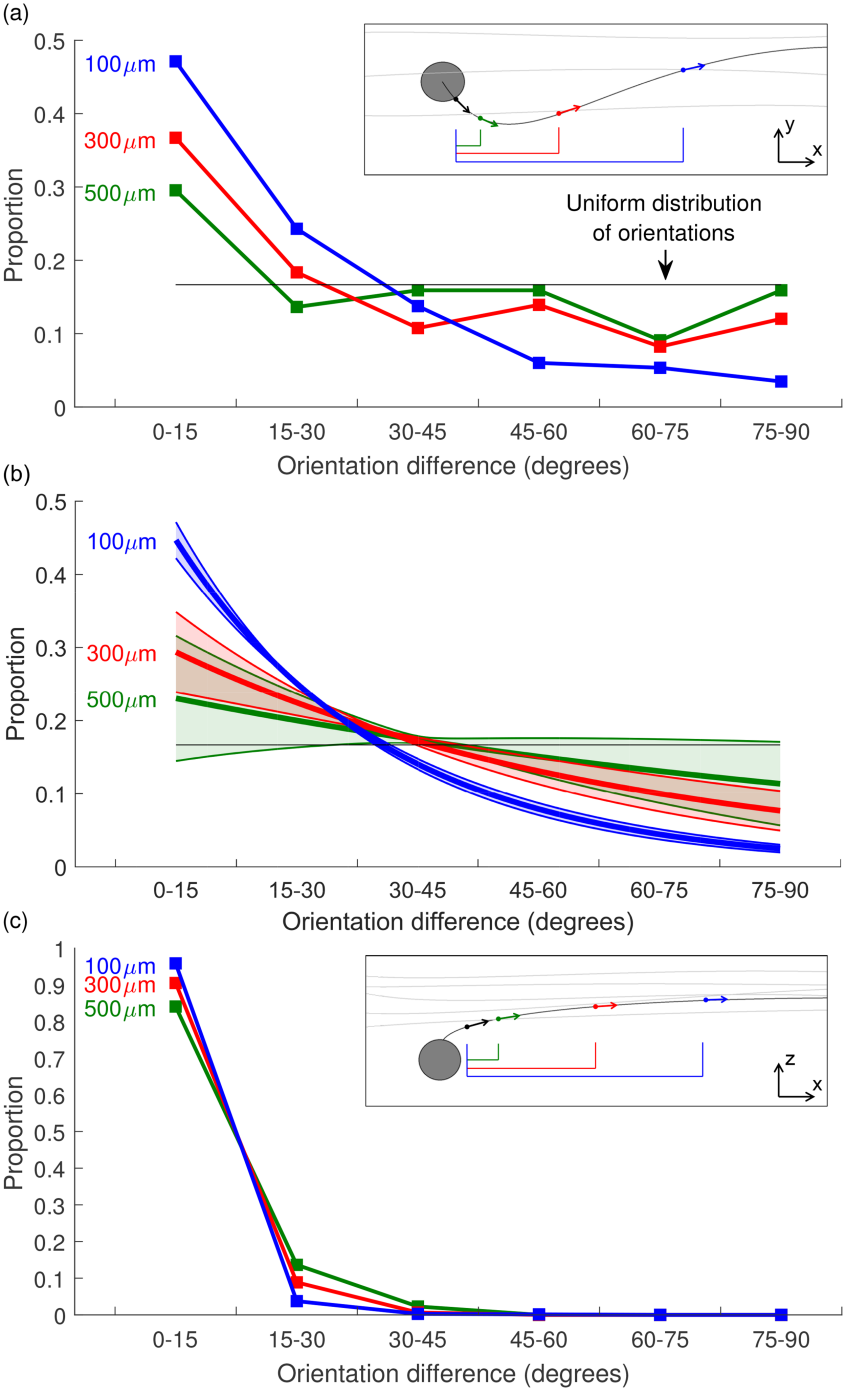
Fiber orientation distributions along the length of the axon. Fiber orientation is calculated relative to the axon initial segment, and determined from all available mammalian retinal ganglion cell reconstructions on NeuroMorpho.org. The distribution of orientations at axonal locations of 100 *μ*m (blue), 300 *μ*m (red), and 500 *μ*m (green) from the soma are shown. (a) Azimuthal (i.e., *x*-*y*) change in orientation between the axon initial segment and more distal axonal locations. (b) Exponential fits to the distributions in (a) with 95% confidence intervals. Statistical analysis of these fits is discussed in the main text. (c) Altitudinal (i.e., *z*) change in orientation between the axon initial segment and more distal axonal locations, with all following an exponential distribution. Insets illustrate planes in which orientations are compared. All orientations are calculated relative to the orientation of the axon initial segment. Due to variation in the length of axon reconstructions, each trace is calculated using a different subset of cells (100 *μ*m—all 749 cells, 300 *μ*m—158 cells, 500 *μ*m—44 cells).

To quantify the distribution of proximal axon orientations in the GCL, we analyzed mammalian RGC reconstructions obtained from the NeuroMorpho.org database [31–42]. At the time of analysis, 749 of the available cell reconstructions included at least 100 *μ*m of the cell’s axon and so were included in our calculations. It is challenging to determine the distribution of RGC subtypes in the sample due to inconsistent classification schemes across studies that span many years and different methodologies. However, we expect that the sample used is representative of the overall RGC population since the majority of cells come from morphological classification studies of the whole population. This is appropriate for the current study as simulations only consider axonal orientation and not cell morphology, so general RGC properties are adequate. Cell morphologies were imported and processed in MATLAB (The Mathworks, Release 2016a) with the third-party TREES toolbox [43]. The change in orientation between the AIS (defined as the segment from 40 *μ*m to 80 *μ*m from the soma [23]) and various locations along the axon was calculated. Fig 2(a) and 2(b) show the proportion of cells with orientation in different ranges. For each cell, the orientation was calculated as the angle between the AIS and the axon, and was measured at axonal locations 100, 300, and 500 *μ*m from the soma. Since each cell has a different length of axon included in the morphological reconstruction (due to differing imaging and sample preparation limitations) different cell sub-populations were available for the distribution at each axonal location. All cells (749) had at least 100 *μ*m of reconstructed axon, 158 cells had at least 300 *μ*m, and 44 had at least 500 *μ*m.

As shown in Fig 2(a), the orientation in the *x*-*y* plane approaches a uniform distribution for locations 500 *μ*m (or more) distal from the soma. This was validated statistically by comparing the goodness-of-fit of uniform and exponential distributions for each location using likelihood-ratio tests. Exponential fits to the distribution in Fig 2(a) are shown in Fig 2(b) with 95% confidence bounds. To account for the fact that fewer cells were available for orientation measurements at 500 *μ*m from the soma, likelihood was calculated for 100 *μ*m (*n* = 749) and 300 *μ*m (*n* = 158) by averaging fits across 1000 random samples of size *n* = 44. This test showed that an exponential fit was more appropriate for the orientation at 100 *μ*m (*p* < 0.001) and 300 *μ*m (*p* = 0.0251), whereas the distribution of orientations at 500 *μ*m was consistent with a uniform fit (*p* = 0.1078). Similar results were found using alternative metrics such as the Akaike information criterion. In contrast, Fig 2(c) shows that there is little change in orientation between the AIS and the distal axon in terms of altitudinal orientation indicating that, beyond the AIS, axons remain predominantly parallel to the surface of the retina. Statistically, an exponential fit was more appropriate than uniform for all distributions in Fig 2(c). Based on the knowledge that fibers in the NFL are approximately parallel to each other at a given location [10, 11, 13], this analysis suggests an approximately circular (but not spherical) uniform distribution is an appropriate approximation for the orientation of AISs in the GCL. A circular distribution will be used in the remainder of this paper.

### Tissue geometry and governing equations

**Fig 3:**
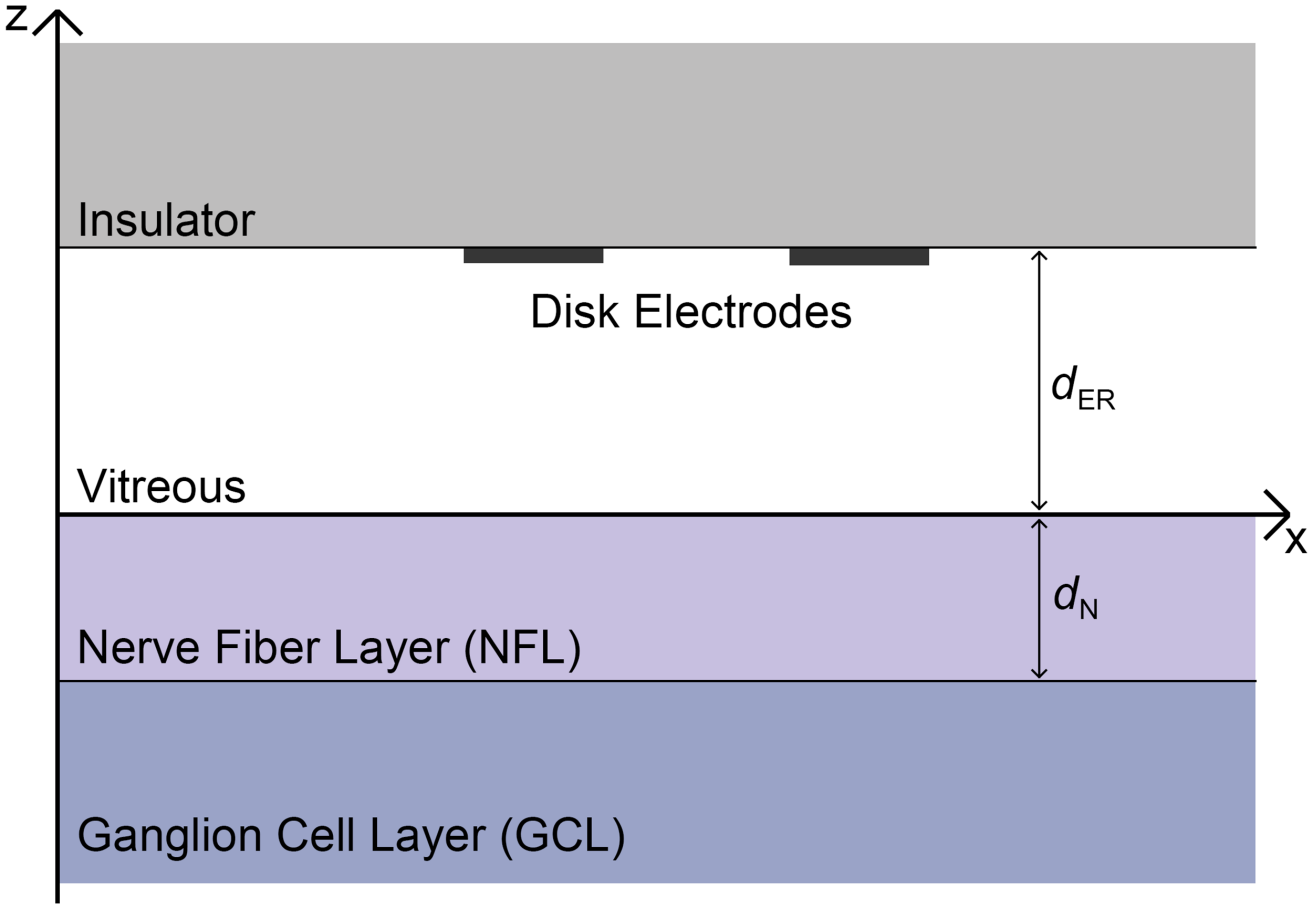
Geometry of the four-layer model of the retina. Modeled layers are the insulator, vitreous, nerve fiber layer, and ganglion cell layer. The insulator is assumed to have zero conductivity and is modeled using a zero flux boundary condition. The GCL is assumed to have infinite extent in the z-direction. The distance from electrodes to the retinal surface and the thickness of the NFL are denoted by *d*_ER_ and *d*_N_, respectively.

The model employed here uses a two-stage volume conductor framework. The first stage models the electric field induced by the stimulating electrodes. The second stage uses the calculated extracellular potential from the first stage as input into a passive neurite model to calculate membrane potential. The present modeling approach uses a four-layer description of retinal geometry for stage one (Fig 3). The modeled layers are the insulating substrate of the electrode array, the vitreous, the nerve fiber layer, and an approximation of the remaining retinal layers, including the ganglion cell layer. The conductivity/admittivity and directional dependence properties of each layer are presented in Table 1. Admittivity is a spatially- and temporally-dependent generalization of conductivity and is the inverse of impedivity, containing both resistive (real) and reactive (imaginary) parts. The anisotropic admittivity of the NFL is incorporated into the complex admittivity kernel provided by the cellular composite model of Meffin et al. [27].

**Table 1:** Conductivity and thickness of modeled layers.

Layer	Directional dependence	Conductivity (S/m)	Thickness (*μ*m)
Insulator	Isotropic	0	Infinite extent
Vitreous	Isotropic	1.78	10-500 (based on placement of electrode array)
NFL	Anisotropic	see S2 Appendix	100 [44–47]
GCL	Isotropic	0.1	Infinite extent

The description of anisotropy/isotropy of each layer is based on a mean-field approximation of the cells that comprise the tissue in that layer. The nerve fiber layer has a markedly anisotropic geometry as it is composed of largely parallel axon bundles. Due to the mix of different cell types and the presence of cell bodies, the GCL and remaining outer retinal layers can be approximated as isotropic on a macro-scale (i.e., isotropic when averaged over some distance). Additionally, it has been shown previously that a combined model of the GCL and outer retinal layers yields approximately equivalent results to a more sophisticated five-layer model of these layers [15].

Due to its (approximately) zero conductivity, the insulator layer is included via a zero current boundary condition applied at the insulator-vitreous interface. Electrodes are modeled as two-dimensional, circular disks lying on this boundary. In this study, we neglect the formation of an electrical double layer at the electrode-vitreous boundary and any associated voltage drop in this region. Experimental and simulation studies have shown that, given the distances between the electrode array and tissue that we consider here, the impact of an electrical double layer is negligible in magnitude as well as being both highly transient and highly spatially localized [18, 48, 49].

The flow of current in the extracellular space in each layer is described by a separate Poisson-type equation, allowing for differing tissue admittivities in each layer, with the driving current delivered by disk electrodes entering as an explicit term on the right-hand-side of the vitreous layer continuity equation:

$$\nabla \cdot J_{V}\left(x,y,z,t\right)=\sum_{i}^{M}\frac{I_{i}\left(t\right)}{\pi q^{2}}g_{q}\left(x-x_{i},y-y_{i}\right)\delta \left(z-z_{i}\right),$$ (1a)

$$\nabla \cdot J_{N}\left(x,y,z,t\right)=0,$$ (1b)

$$\nabla \cdot J_{G}\left(x,y,z,t\right)=0,$$ (1c)

where subscripts V, N, and G associate quantities with the vitreous, NFL, and GCL, respectively. **J**_*α*_ is the extracellular current density in layer *α* and each set of (*x*_*i*_, *y*_*i*_, *z*_*i*_) represents the three-dimensional location of one of the *M* electrodes. Each electrode has radius *q* and stimulus current waveform *I*_i_(*t*). The function *g*_*q*_(*x*, *y*) is the unit circular step function of radius *q* in the *x*-*y* plane and *δ*(*z*) represents the Dirac delta function. ∇ = [*∂*/*∂x*; *∂*/*∂y*; *∂*/*∂z*] is the differential operator. For this model, each layer boundary is approximated by an infinite flat plane parallel to the *x*-*y* plane, so that the set of electrode heights, *z*_i_, are equal. Furthermore, if the origin is fixed on the vitreous-NFL boundary, then *z*_i_ is equivalent to the electrode-retina separation distance, *d*_ER_.

A generalized form of Ohm’s Law is used to describe extracellular current density and potential, which is governed by each layer’s admittivity kernel. This admittivity kernel incorporates the dependence of the extracellular current density on the electric field at previous times and at remote locations in the extracellular space. These atypical dependencies arise due to the passage of current across the cellular membrane and through the intracellular space. The relationship between extracellular potential and current density is described by

$$\begin{array}{c} \begin{array}{cc} J_{\alpha } & =-\frac{1}{4\pi^{2}}\xi_{\alpha }\left(x,y,z,t\right)*\nabla \phi_{\alpha }\left(x,y,z,t\right)\\ & =-\frac{1}{4\pi^{2}}\underset{r^{\prime }\;t^{\prime }}{\;\int \int \;}\xi_{\alpha }\left(r^{\prime },t^{\prime }\right)\nabla \phi_{\alpha }\left(r-r^{\prime },t-t^{\prime }\right)dr^{\prime }dt^{\prime }, \end{array} \end{array}$$ (2)

where ξ_*α*_ is the 3x3 admittivity kernel and *ϕ*_*α*_ is the extracellular potential of layer *α* ∈ {V, N, G}. In the most general case, where ξ_*α*_ varies in three spatial dimensions and time, * represents a convolution over three spatial dimensions and time. For brevity, the spatial coordinates (*x*, *y*, *z*) have been represented by the vector **r** in the integral expression for the convolution.

For layers with infinite extent in the *x*- and *y*-directions, as in the present model, boundary conditions are specified at the layer boundaries:

$$\phi_{G}{|}_{z=-\infty }=0,$$ (3a)

$$\phi_{N}{|}_{z=-d_{N}}=\phi_{G}{|}_{z=-d_{N}},$$ (3b)

$$\phi_{V}{|}_{z=0}=\phi_{N}{|}_{z=0},$$ (3c)

$$J_{N_{z}}{|}_{z=-d_{N}}=J_{G_{z}}{|}_{z=-d_{N}},$$ (3d)

$$J_{V_{z}}{|}_{z=0}=J_{N_{z}}{|}_{z=0},$$ (3e)

$$J_{V_{z}}{|}_{z=d_{\text{ER}}+d_{\text{EI}}}=0.$$ (3f)

These boundary conditions ensure that the described system has finite energy (Eq (3a)), that current density and voltage vary continuously across layer boundaries (Eqs (3b)–(3e)), and that no current can flow into the insulating substrate (Eq (3f)).

Since the current sources are at the same *z*-location as the insulator’s zero current condition, we initially define the geometry such that the insulator is separated from the electrodes by some distance, *d*_EI_. This is eliminated subsequently by computing the limit from above as *d*_EI_ goes to zero. As a result, we maintain a zero current boundary condition at the insulator, except for current coming out of the electrode sources, which are modeled as explicit current sources in Eq (1a).

Solution of the system of elliptic partial differential equations defined by Eqs (1) and (2) using layer boundary conditions (3) yields expressions for the extracellular potential in each layer. To find a closed-form solution to this system, we first assume that within each layer tissue admittivity is independent of *z*, reducing the above four-dimensional convolutions to three dimensions. Fourier domain approaches are then applied to reduce the convolutions shown in Eq (2) to multiplications. Eqs (1) and (2) are transformed into the Fourier domain with respect to *x*, *y*, and *t*. The system is then represented by a system of partial differential equations for which an analytic solution exists of the form

$${\hat{\phi }}_{V}=A_{1}e^{-\eta_{V}z}+A_{2}e^{\eta_{V}z}+\sum_{i}^{M}\frac{m_{i}}{2\eta_{V}}e^{-\eta_{V}\left|z-z_{i}\right|},$$ (4a)

$${\hat{\phi }}_{N}=B_{1}e^{-\eta_{N}z}+B_{2}e^{\eta_{N}z},$$ (4b)

$${\hat{\phi }}_{G}=C_{1}e^{-\eta_{G}z}+C_{2}e^{\eta_{G}z},$$ (4c)

where *A*_1_, *A*_2_, *B*_1_, *B*_2_, *C*_1_, and *C*_2_ are constants of integration and remaining parameters are defined in S1 Appendix, along with a detailed derivation of this solution. The hat symbol (^) indicates the Fourier transform of the specified quantity with respect to *x*, *y*, and *t*. Quantities *m*_i_ and *η*_*α*_ are defined in terms of the Fourier domain pairs of *x*, *y*, and *t*. The form of *m*_i_, shown in S1 Appendix, defines both the geometry of the electrodes (the spatial Fourier transform of a disk), and the current stimuli (the temporal Fourier transform of a biphasic square pulse). Stage one of the volume conductor model is completed by determining appropriate values for the admittivity or conductivity of each of the modeled layers and is presented in S2 Appendix.

### Neurite equations

Stage two of the cellular composite model involves the calculation of the passive membrane potential in the neurite of interest in either the NFL or the GCL. This is achieved using the neurite equations of Meffin et al. [25], which provide expressions for membrane activation due to modes of current flow that are both longitudinal (*V*_m,L_) and transverse (*V*_m,T_) with respect to the fibers. Expressions for the membrane potential along a single fiber in a fiber-bundle with orientation parallel to the y-axis (as in the NFL) are supplied in the *x*, *y*, *t*-Fourier domain by the cellular composite model,

$${\hat{V}}_{m,L}\left(k_{y},\omega ;k_{x},z\right)=-\frac{k_{y}^{2}\lambda_{V}^{2}\left(\omega \right)}{1+k_{y}^{2}\lambda_{V}^{2}\left(\omega \right)}{\hat{\phi }}_{\alpha }\left(k_{y},\omega ;k_{x},z\right),$$ (5a)

$${\hat{V}}_{\text{m},\text{T}}\left(k_{y},\omega ;k_{x},z\right)=-b\sqrt{[}-k_{x}^{2}{\hat{\phi }}_{\alpha }{\left(k_{y},\omega ;k_{x},z\right)}^{2}+{\left(\frac{\partial {\hat{\phi }}_{\alpha }\left(k_{y},\omega ;k_{x},z\right)}{\partial z}\right)}^{2}],$$ (5b)

where *k*_*x*_, *k*_*y*_, and *ω* are the Fourier transform pairs of *x*, *y*, and *t*, respectively. ${\hat{\phi }}_{\alpha }\left(k_{y},\omega ;k_{x},z\right)$ is the Fourier domain representation of extracellular potential along the neurite axis for a straight neurite oriented parallel to the *y*-axis at a point (*k*_*x*_, *z*). λ_V_(*ω*) is the frequency-dependent electrotonic length constant for voltage boundary conditions and is defined in S2 Appendix. Eq (5a) is a Fourier domain representation of the cable equation for extracellular stimulation and indicates the dependence of *V*_m,L_ on the second spatial derivative of the extracellular potential in the direction of the neurite, known as the activating function [30]. Here, the activating function is represented in the Fourier domain as $-k_{y}^{2}\phi_{\alpha }$.

S3 Appendix provides an extension of expressions for the longitudinal and transverse components of the membrane potential to straight neurites of arbitrary *x*-*y* orientation, allowing for analysis of fibers in both the NFL (fibers with parallel orientation) and GCL (fibers with arbitrary *x*-*y* orientation).

### Calculating membrane potential thresholds

Several studies have examined the difference in excitability of the AIS and the AOP or axon bundles [10, 23]. Electrical stimulation experiments conducted by Fried et al. [23] found that a high-density sodium channel band exists in the RGC AIS. They confirmed the existence of the high-density sodium channel band using both electrical recording of cell responses and immunochemical cell staining with an antibody stain of ankyrin-G that colocalizes with sodium channels. When compared to the soma and the distal axon or AOP, the high-density sodium channel band at the AIS had a greater sensitivity to electrical stimulation. Heightened excitability at the AIS has also been demonstrated in the central nervous system more generally. In the cortex, this has been shown experimentally for pyramidal cells via investigations of the site of action potential initiation [50, 51], action potential initiation thresholds [51, 52], and sodium channel density [51].

As ion channels are not modeled in the present model, the difference in excitability of the AIS and AOP is instead captured by adjusting the threshold membrane potential at each location. Threshold potential values for the AIS and AOP have been determined from simulations that replicate the experimental procedures of Fried et al. [23]. By matching the experimental electrode geometry, electrode location, neurite orientation, nerve fiber layer thickness, and stimulation frequency, Fried’s experimentally-determined threshold stimulus currents were mapped to corresponding threshold membrane potentials in the computational model presented here. A recent study by Chichilnisky and colleagues [10] has also demonstrated conditions under which activation of passing axon bundles and the AIS occurs, however we were not able to reproduce these experiments in simulations without cell morphologies and the precise three-dimensional location of the stimulating electrodes relative to the different axon locations.

**Fig 4:**
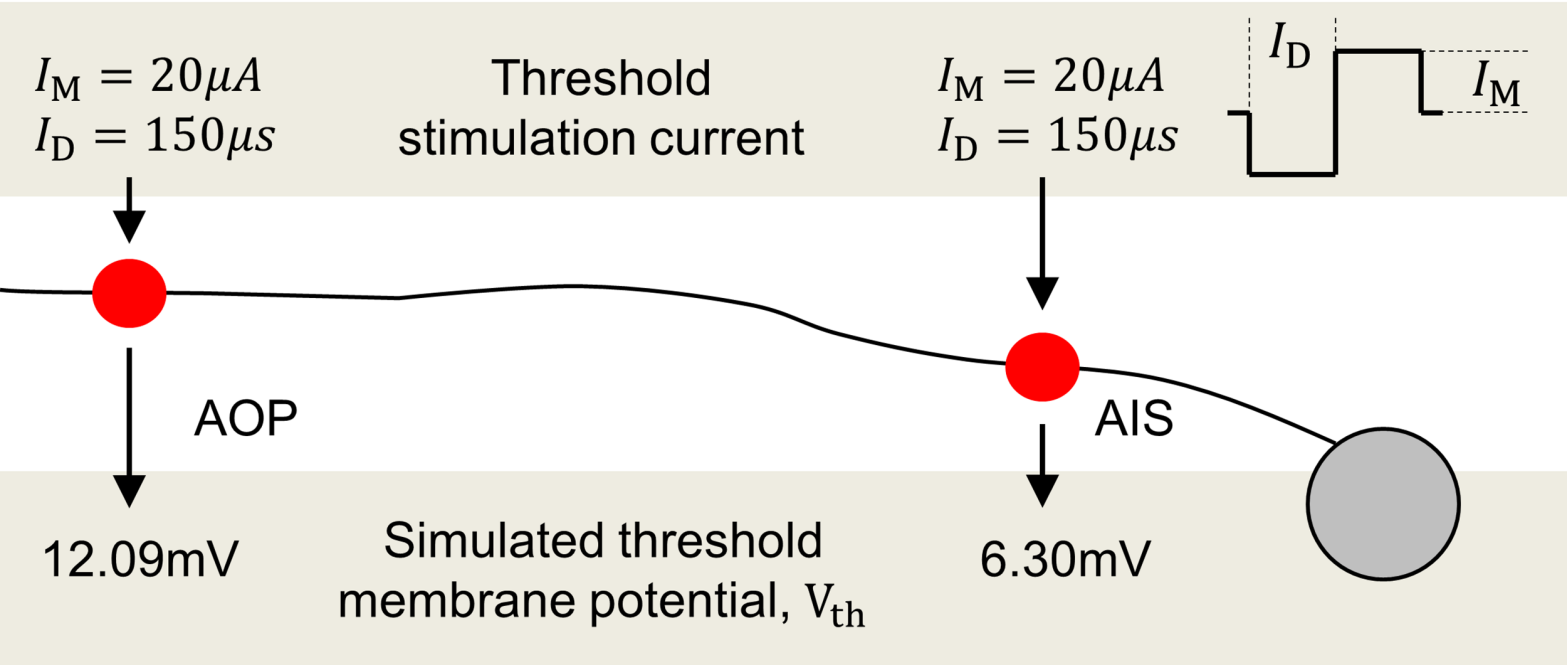
Simulation of experiments from Fried et al. [23]. The current waveforms used for stimulation and the maximum simulated membrane potential responses are shown. The maximum simulated membrane potential for each simulation corresponds to the membrane threshold, *V*_th_, for that location in the axon.

In order to design simulations that most closely match the experimental methodology, nerve fiber layer thickness, *d*_N_, was set to 25 *μ*m, appropriate for a rabbit retina. A single electrode with a radius, *q*, of 15 *μ*m was used to deliver a single cathodic-first, biphasic pulse from a location 25 *μ*m from the surface of the retina (*d*_ER_ = *z*_i_ = 25 *μm*). Stimulus pulse amplitudes were chosen to approximately match the stimulus threshold levels reported by Fried et al. [23]. Experimentally reported stimulus current thresholds for the AIS and the distal axon were then used as pulse amplitudes in simulations, which are illustrated in Fig 4. These stimulus current thresholds happened to be approximately 20 *μ*A for both the initial and distal axon. This is due to the fact that, although the AIS has a markedly lower threshold membrane potential (not a lower *stimulus* current threshold), the AOP is closer to the stimulating electrode. In this analysis, the AIS was assumed to be 5 *μ*m below the surface of the GCL and the AOP was assumed to be centered in the NFL, 12.5 *μ*m below the retinal surface. The experimental procedure of Fried et al. used narrow conical electrodes with no backing insulator and so the insulator layer was removed in these simulations. Using these parameters, the maximum simulated membrane depolarization achieved in an axon below the electrode corresponded to the relevant membrane threshold. Membrane thresholds were 12.09 mV and 6.30 mV above resting membrane potential for the AOP and the AIS, respectively. Note that this analysis used a NFL thickness of 25 *μ*m appropriate for the rabbit retina whereas human NFL thickness is in the order of 100 *μ*m, and is considered for the remainder of the paper. The assumption is that the ratio of membrane thresholds between the AIS and AOP is suitably consistent across vertebrate species [53]. To ensure the present result is robust to this assumption and to the calculated threshold membrane potential values, a sensitivity analysis was conducted to assess the dependence of the final simulation results, presented in the Results section.

### Simulation methods

Simulations of a wide range of electrode and current waveform variations were conducted in MATLAB. All computations of induced extracellular potential and membrane potential were first calculated in the spatial and temporal frequency domains using Fourier domain solutions of the modeled system. The frequency representation of the longitudinal and first transverse components of a neurite’s or a volume’s membrane potential were summed together prior to calculating the inverse Fourier transform, yielding the final membrane potential. The solution to the system described above is found in the Fourier domain with respect to the x and y spatial dimensions and the temporal dimension. Due to this, each simulation required the calculation of extracellular and membrane potential in an entire spatial plane and for the full temporal extent of the simulation before the inverse Fourier transform was calculated.

This analysis considered only direct cell responses and neglected the effect of retinal networks. As such, the output of the passive membrane potential model was compared to pre-calculated membrane thresholds for the AIS and AOP to determine corresponding levels of activation.

To determine the proportion of fibers activated at a given location within the retina, the activity of fibers with an appropriate range of orientations in the *x*-*y* plane was first calculated and then combined in a weighted sum, where the weights were sampled from an assigned distribution of orientations. For locations in the NFL, a single parallel orientation was assumed, whereas, for the GCL, a uniform distribution of orientations was applied in the *x*-*y* plane, as validated in Fig 2.

We describe and analyze the results of simulations of straight cylindrical neurites embedded in the modeled four-layer retinal structure. For all simulations, 100 *μ*m diameter disk electrodes were used unless stated otherwise. For simulations of multi-electrode stimulation, electrodes were arranged in a regular grid with 200 *μ*m center-to-center spacing between electrodes. Unless otherwise stated, stimuli used were cathodic-first, biphasic pulses with a pulse width of 200 *μ*s. In addition, for all multi-electrode simulations, equal currents were applied to each electrode.

Due to the importance of anisotropy in the NFL, which depends on the thickness of the NFL, it is required that human retinal geometries be used in simulations. In general, the required model parameters are not expected to be specific to the animal being simulated. The RGC axon radius, *a*, has been shown experimentally to be consistent across several animal and human studies [31–42, 54, 55]. All relevant model parameter values used are presented in Table 2. Note that since the model includes a description of both the intracellular and extracellular components of the relevant retinal layers, we use the extracellular resistivity associated with the extracellular medium, and not the *effective* resistivity of the tissue, which is influenced by cells and the extracellular medium. To compare the activation of AOPs in the NFL and AISs in the GCL, we considered characteristic axons located just (*z* = 10 *μ*m) below the surface of their respective retinal layer, as structures at these locations are most sensitive to epiretinal stimulation.

**Table 2:** Model parameter values, unless stated otherwise.

Parameter	Description	Value	Reference
*a*	Neurite radius	0.47 *μ*m	[31–42, 54, 55]
*d*	Extracellular sheath width	30 nm	[56]
*ρ*_i_	Intracellular resistivity	0.7 Ωm	[57, 58]
*ρ*_e_	Extraellular resistivity	0.7 Ωm	[57, 58]
*R*_m_	Membrane resistance	1 Ω m^2^	
*C*_m_	Membrane capacitance	0.01 F/m^2^	
*d*_N_	Nerve fiber layer thickness	100 *μ*m	[44–47]
*d*_ER_	Electrode-retina separation	10-500 *μ*m	
*q*	Radius of disc electrodes	50 *μ*m	

An implementation of the computational model used in this paper is hosted publicly on GitHub (https://github.com/timesler/FourLayerRetinalModel-Esler2017).

## Results

### Analysis of tissue anisotropy

**Fig 5:**
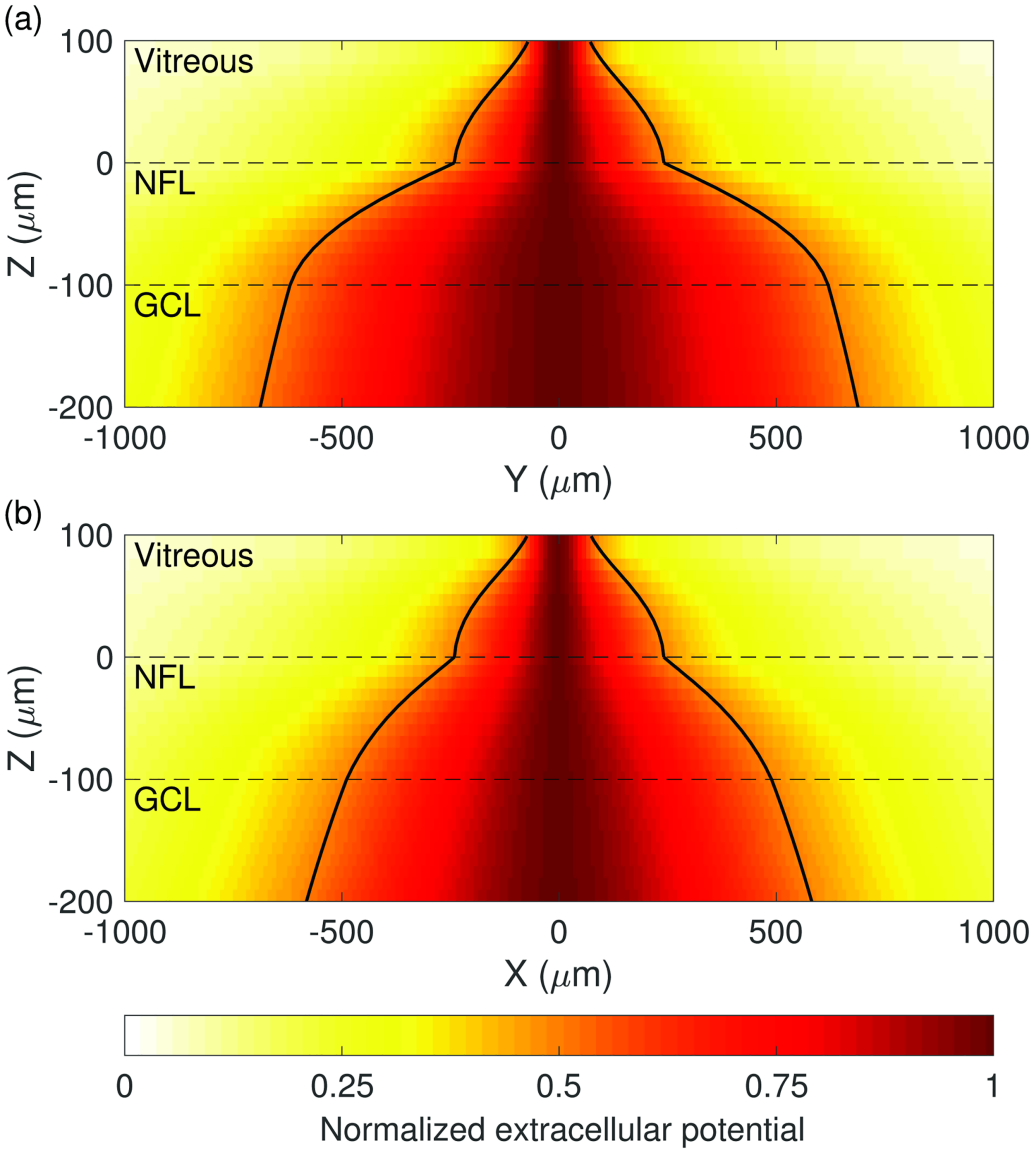
Normalized spread of extracellular potential with distance from a stimulating electrode. Spread is shown in (a) the *y*-*z* plane, parallel to the orientation of AOPs, and in (b) the *x*-*z* plane, perpendicular to the orientation of AOPs. The simulated extracellular potential at each *z*-slice is normalized to the range [0, 1] by subtracting the minimum and scaling the maximum per slice to 1. This is done for illustrative purposes due to the rapid fall-off of extracellular potential with increasing distance from the electrode. Contour lines indicate the full-width at half-maximum potential. Stimulation is with a single electrode located 100 *μ*m above the retinal surface at the origin in the *x*-*y* plane. Dashed lines indicate layer boundaries.

A prerequisite for tissue orientation-dependent activation of RGCs is that tissue anisotropy translates into anisotropic spread of extracellular potential. Fig 5 shows the normalized spread of current versus depth in the retina for planes parallel and perpendicular to the orientation of fibers in the NFL. Anisotropy of current spread is demonstrated, with a 760 *μ*m increase in the half-width at full-maximum extracellular potential across the NFL in the *y*-*z* plane (parallel to the orientation of fibers in the NFL) compared to 500 *μ*m in the *x*-*z* plane. This represents a 1.52x greater spread of extracellular potential in the direction of passing fibers.

### Comparison of one-, two-, and four-electrode configurations

**Fig 6:**
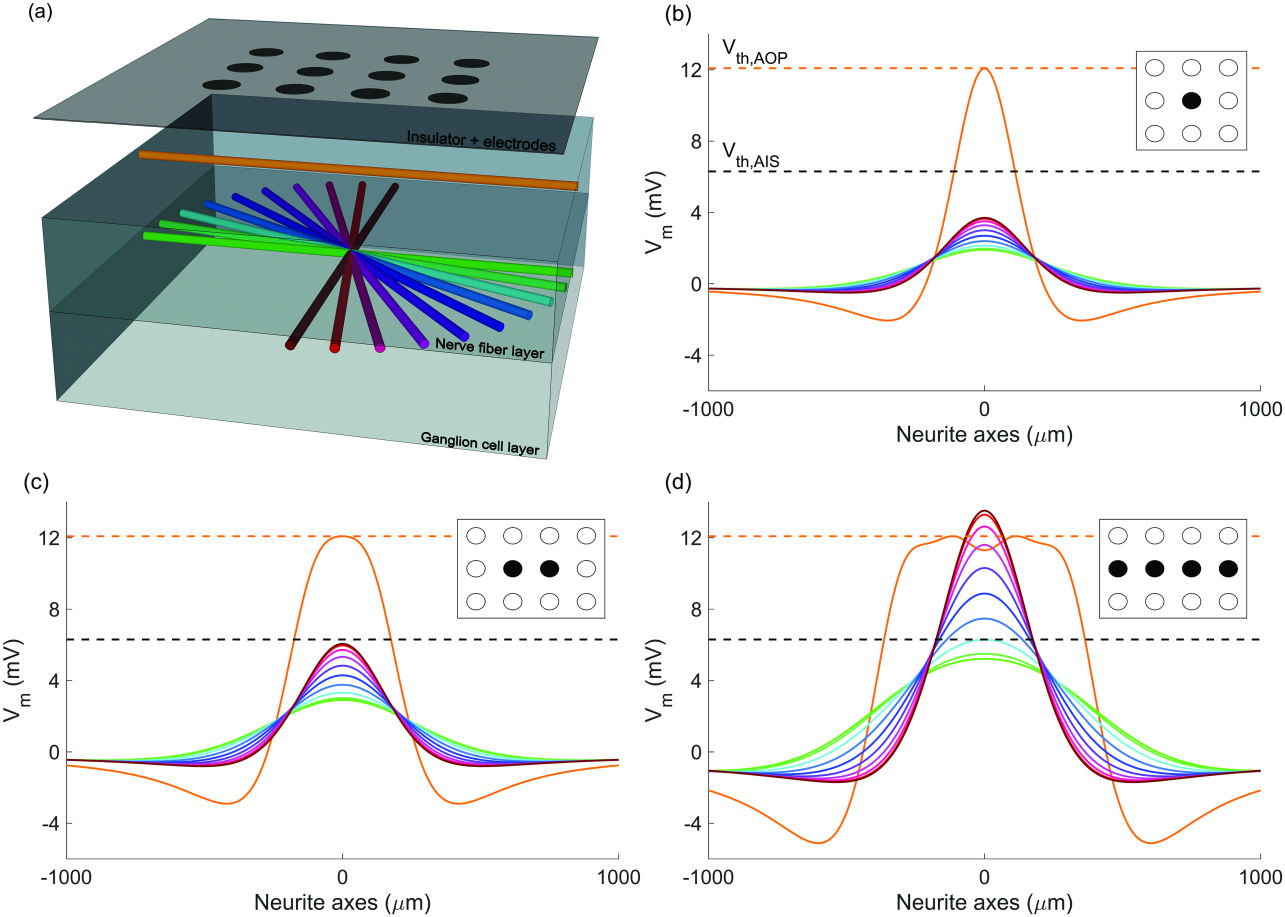
Geometry and simulated membrane potentials for axons of passage and axon initial segments at a variety of *x*-*y* orientations. (a) Four-layer model geometry showing the electrode array, an example of a parallel axon of passage (orange), and the neurite orientations considered in the ganglion cell layer (green-brown). Membrane potential at the end of the cathodic phase is shown along the axes of the neurites being simulated for configurations of (b) one, (c) two, and (d) four electrodes aligned with the axon of passage. Dotted lines represent membrane thresholds for axons of passage (orange) and axon initial segments (black). Stimulus currents have been chosen such that they drive the axon of passage precisely to its threshold level. Colors in (b)-(d) indicate corresponding neurites in (a).

To establish a basis for fiber orientation-dependent activation in the retina, simulations were run to determine the activation for 1) a single characteristic AOP and 2) AISs with a range of sampled azimuthal orientations. The geometry of these simulations is represented in Fig 6(a). Fig 6(b), 6(c) and 6(d) show the membrane potential resulting from stimulation with one, two, and four electrodes, respectively, for fibers with orientations illustrated in Fig 6(a). Fig 6(b) highlights the influence of the NFL anisotropy on the activation of GCL fibers of different orientations, with fibers orientated perpendicularly to the AOP experiencing 1.9 times the depolarization of parallel fibers. Fig 6(b) also highlights the problem being addressed by this research: although the target AISs in the GCL are more excitable due to the presence of the high-density sodium channel band (represented here by a lower threshold), the proximity of the NFL to stimulating electrodes results in the preferential activation of AOPs.

Fig 6(c) and 6(d) provide an initial assessment of the combined influence of tissue anisotropy and electric field orientation on activation of AOPs and AISs. The work of Rattay and Resatz [11] indicated that the activation of a passing fiber may be limited by controlling the way in which the induced electric field changes along the length of that fiber. Hence, simulations have been designed that recruit a number of electrodes aligned with the direction of the considered AOP. As can be seen from Fig 6, the level of AIS versus AOP activation increases markedly as the number of electrodes increases. With four electrodes, it is possible to activate 78% of AIS fibers before activation of the overlying layer. When compared to Fig 6(b), there is a consistent increase in the relative activation of perpendicular (green) and parallel (brown) AISs in the GCL for two- and four-electrode configurations. For comparison, the ratios of perpendicular to parallel AIS activation are 1.9, 2.1, and 2.5 for one, two, and four electrodes, respectively.

### Effect of pulse duration and electrode-retina separation

**Fig 7:**
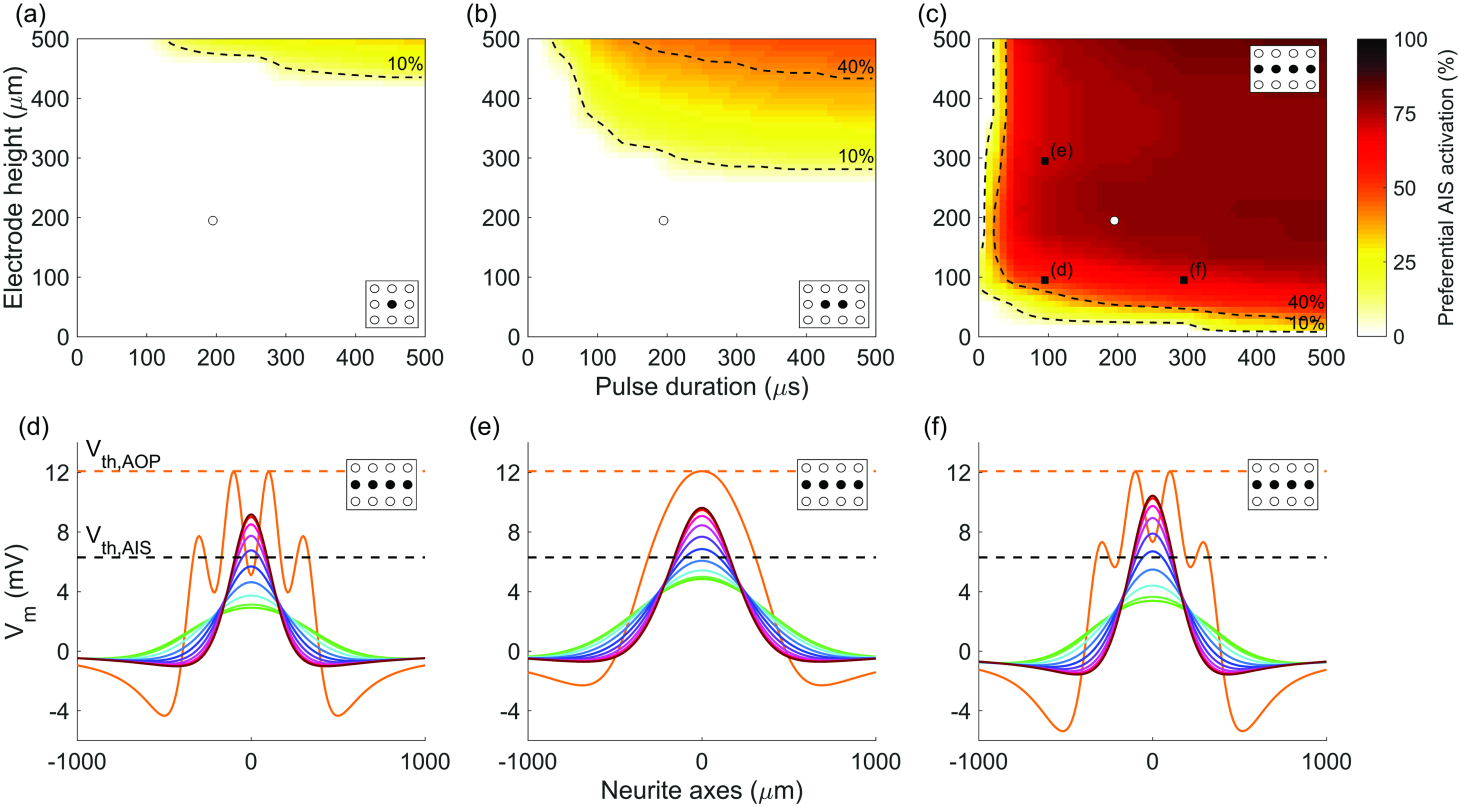
Proportion of axon initial segment orientations preferentially activated for different electrode-retina separations (*d*_ER_) and pulse durations. Heat maps indicate the proportion of axon initial segments activated at a lower stimulus current than any fibers in the nerve fiber layer for (a) one-, (b) two- and (c) four-electrode configurations (aligned with the axon of passage). Regions of low (<10%), medium (10-40%), and high (>40%) stimulation selectivity are separated by dotted contours. White markers indicate the parameters used in Fig 6, and black markers indicate the parameters used for subplots (d), (e) and (f), which show examples of simulated membrane potentials for axons of passage and axon initial segments. Colors in (d)-(f) correspond to those in Fig 6(a).

A parameter sweep was conducted over pulse duration and electrode-retina separations. For each set of parameters, simulations were run to compare the membrane activation of parallel neurites in the NFL and neurites with a range of rotated orientations in the GCL. For both the single orientation in the NFL and the range of simulated fiber orientations in the GCL, membrane potential was calculated for fibers across the full plane at the appropriate retinal depth. Under the assumption that the *xy*-orientation of AISs is described by a uniform distribution, the proportion of preferentially activated AIS fiber orientations was determined. This is illustrated in Fig 7, which shows the level of preferential activation achieved for a variety of stimulation parameter combinations. In this analysis, preferential activation is defined as when the membrane potential of an AIS is driven to its threshold potential at a lower stimulus current than is required to drive *any* AOP to threshold.

Fig 7(a) highlights the challenge of achieving preferential activation of the GCL using single-electrode stimulation. Only very small levels of selectivity are obtained even with the most favorable stimulation parameters (large electrode height and pulse duration). A dramatic increase can be seen in the range of stimulation parameters at which preferential activation is achieved when moving from the one- or two-electrode configurations to four electrodes.

A comparison of the membrane potential induced by four-electrode stimulation with small and large electrode-retina separation can be seen by comparing Fig 7(d) and 7(e). A clear effect is that, due to the smoothing effect of increased current spread with greater electrode-retina separation, the AOP membrane potential along the axon has a much smoother shape for electrodes positioned further from the retina. Less intuitively, larger separation distances result in increases in preferential activation of the GCL. This is due to the increased opportunity for summation of currents originating from adjacent electrodes. Similarly, as can be seen in Fig 7(d) and 7(f), increases in pulse duration also result in increases in preferential activation of the GCL. Importantly, however, the overwhelming majority of the change in preferential activation occurs for pulse durations of less than 50 *μ*s and separation distances of less than 100 *μ*m. Although preferential activation increases with pulse duration, above 50 *μ*s the increases become negligible, so a duration of 50 *μ*s may be preferred due to the lower power required.

### Sensitivity of results to threshold membrane potential

Due to the dependence of these findings on the chosen threshold values at the AIS and AOP, a sensitivity analysis was performed. Fig 7(c) presents four-electrode stimulation results obtained using an AOP to AIS membrane potential threshold ratio of approximately 2 (12.09 mV versus 6.30 mV), as determined from simulations of experiments conducted by Fried et al. [23]. Identical four-electrode analyses were conducted using ratios of 1.5 and 1. Each case was compared by calculating the proportion of the plot area for which greater than 10% preferential AIS activation was achieved. This yields 93% for a ratio of 2 as shown in Fig 7(c), 80% for a ratio of 1.5, and 24% for a ratio of 1. As expected, increasing the relative membrane threshold of the AIS decreases its propensity for preferential activation. This decrease is modest for a more conservative ratio of 1.5. Even with a ratio of 1, corresponding to equal membrane potential thresholds at the AIS and AOP, a level of preferential activation is achievable with four-electrode stimulation. This is due to the beneficial influence of electrode alignment and tissue anisotropy on the shape of the induced electric field. The resultant shape tends to more readily stimulate fibers with an orientation perpendicular to those in the NFL, causing preferential activation of some fibers in GCL target region.

### Performance of simultaneous four-electrode stimulation

An important assessment of these results is how the increase in preferential activation of the GCL affects key clinical performance metrics, such as the required stimulus charge density and the spatial selectivity of activation, which is measured here using activation radius. In the following analysis, GCL activation level is defined as the percentage of AIS orientations that are activated (depolarized to above membrane threshold) given a specific stimulus. This percentage is taken at the point in the plane of analysis that is maximally activated, which in all simulated examples is centered with respect to the activated electrodes. Activation radius is used to show the width of the region that is activated by a given stimulus, which will directly affect the resolution achievable with an implanted device and is defined as the radius of the smallest circle that encloses all areas with non-zero activation.

**Fig 8:**
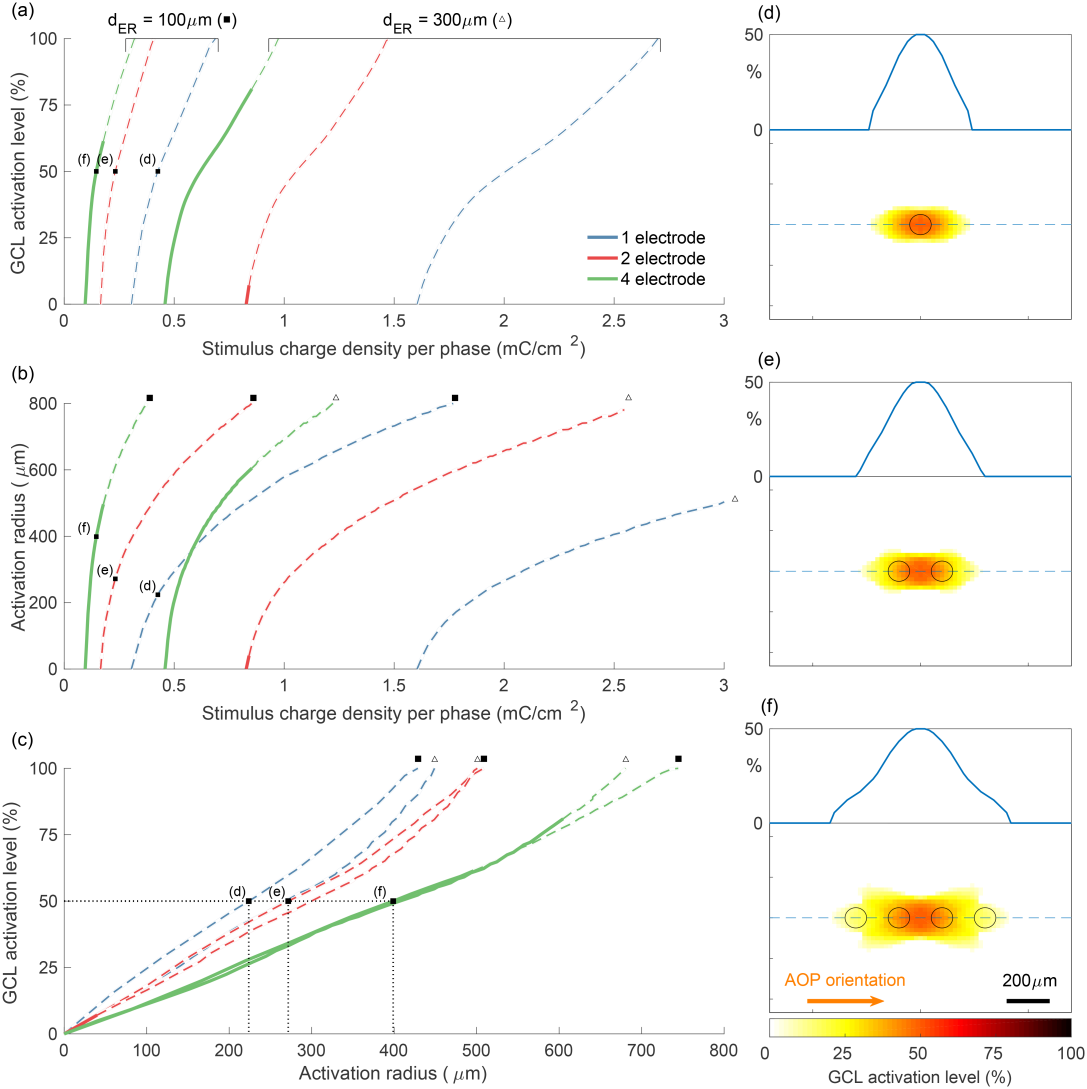
Performance of different electrode configurations with respect to GCL activation level, required stimulus charge density per phase, and radius of activation. (a) The proportion of AIS orientations activated vs. stimulus charge density for various electrode configurations and electrode-retina separation distances, *d*_ER_. (b) The radius of the activated region vs. stimulus charge density per phase. (c) The relationship between activation radius and activation level. Stimulation strategies analyzed in (a)-(c) include one-, two-, and four-electrode configurations, as well as separation distances of 100 *μ*m (filled square, ■) and 300 *μ*m (unfilled triangle, Δ). Solid and dashed regions in (a)-(c) represent configurations that result in preferential activation of AISs and preferential activation of AOPs, respectively. Labeled points in (a)-(c) correspond to the examples plotted in (d)-(f), which show the spread of GCL activation in the *x*-*y* plane when stimulus charge is set to acheive maximum GCL activation of 50%. Dashed blue lines in (d)-(f) correspond to one-dimensional insets. All simulations used a pulse phase duration of 200 *μ*s, with amplitudes indicated in terms charge density per phase.

Fig 8(a) shows the relationship between stimulus charge density and GCL activation level and how this relationship changes with electrode configuration and electrode-retina separation distance, *d*_ER_. As expected, to achieve an equal level of activation for more distant electrodes, greater stimulus charge is required. Fig 8(b) shows the variation in activation radius with stimulus charge density and Fig 8(c) shows the correspondence between activation level and activation radius in the GCL. In each of Fig 8(a)–8(c), dashed curve regions indicate undesirable stimulation configurations, in which AOPs are activated preferentially or in addition to AISs. In terms of isolating the optimal stimulus level, it is important to consider whether this will result in co-activation of passing axons (as indicated by dashed regions), the level of activation achieved in the GCL, and the resulting radius of activation in the GCL. To facilitate comparison of the spread of activation in the GCL induced by one-, two-, and four-electrode configurations, two-dimensional maps of activation in the *x*-*y* plane are shown in Fig 8(d)–8(f), along with the locations of the stimulating electrodes. Importantly, despite utilizing four times the number of electrodes, the activation radius at a given activation level for the four-electrode configuration is typically less than 200% of the activation radius for one electrode, as indicated by dotted lines in Fig 8(c). Furthermore, for four electrodes, the required stimulus charge density is reduced to 0.34x of that required for one electrode.

### Non-ideal electrode array placement

In practice, electrodes are unlikely to be ideally aligned with passing axons as in Fig 8(f). This is due to both the placement of the implanted device and the curvature of passing axons as they pass under the electrode array. To test the application of the multi-electrode stimulation strategy for non-ideal electrode placement, several more challenging geometries were simulated. In each case, the electrodes recruited for stimulation were chosen to represent the most logical extension of the ideal four-electrode configuration presented above and the electrodes were stimulated with equal current.

**Fig 9:**
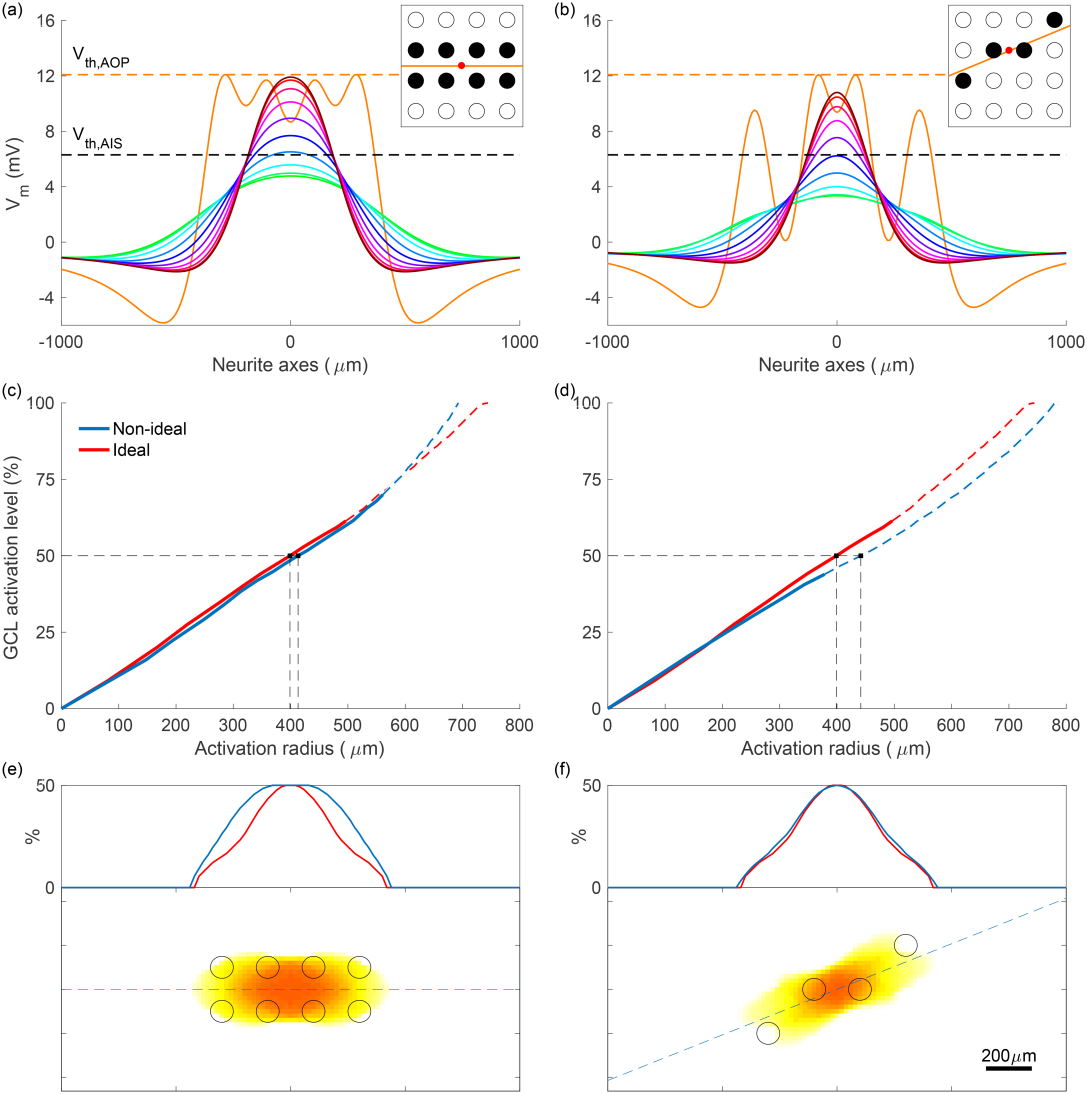
Preferential stimulation for two non-ideal electrode array placements. (a)-(b) Membrane potential along neurite axes for axons of passage and axon initial segments, with stimulus current chosen to maximally activate initial segments without activating any passing axons. Colors correspond to those in Fig 6(a), with green parallel to axons of passage and brown perpendicular. Insets describe the geometry of each simulation, indicating target region (red), electrodes used (black), and the orientation of axons of passage (orange). (c)-(d) Ganglion cell layer activation level vs. activation radius for non-ideal and ideal (as in Fig 8(f)) geometries. Transitions from solid to dashed lines represent the transitions from axon initial segment to axon of passage preferential activation. (e)-(f) The spread of ganglion cell layer activation in the *x*-*y* plane. The dashed blue line corresponds to the one-dimensional inset. Colors are mapped according to the color bar in Fig 8. All simulations used a pulse phase duration of 200 *μ*s and electrode-retina separation of 100 *μ*m.

Fig 9 shows an assessment of two such geometries: one where the target for stimulation is centered between four electrodes and another where the target for stimulation is centered between two electrodes and with a non-parallel axon of passage orientation of 22.5 degrees, as shown in the insets in Fig 9(a) and 9(b). For the former case, stimulation current was delivered by eight electrodes in total. Another obvious choice of AOP orientation to analyze is 45 degrees. However, because this orientation aligns with diagonal rows of electrodes, the outcome was very similar to the ideal, 0 degree case and so has been omitted here.

The resulting membrane potentials along the axis of an AOP and AISs with varied orientations are presented in Fig 9(a) and 9(b). For each configuration, preferential activation of AISs was achieved, with 70% and 44% of AIS orientations being activated at lower stimulus currents than any AOPs for the eight- and four-electrode configurations shown, respectively (compared to 61% for the ideal four-electrode configuration). As shown in Fig 9(c) and 9(d), the relationship between GCL activation and activation radius is comparable with that of the ideal configuration. Finally, the *x*-*y* activation maps in Fig 9(e) and 9(f) indicate only modest increases in the spread of activation when compared to the ideal case. Simulations of several additional edge cases such as off-center target region, are presented in S1 Fig

## Discussion

### Key factors influencing preferential retinal activation

The observed dependence of activation on neurite orientation is a result of several competing factors. The dominant orientation of axons in the NFL results in highly anisotropic spread of extracellular potential under stimulation. As a result, the orientation of fibers in the GCL with respect to this anisotropy influences membrane potential. The overall probability of eliciting a response selectively in the GCL and not in the NFL then depends on the relative influence of fiber rotation, membrane threshold, and fiber depth.

As shown by Fig 5, current spreads through the NFL much more readily in the direction of the overlying fiber tracts than it does perpendicularly to them. This leads to a more rapid change in extracellular potential when moving away from stimulating electrodes in the direction perpendicular to the AOPs. This, in turn, results in the directional spatial derivatives of extracellular potential being greater in this perpendicular direction, leading to maximal activation of AISs with perpendicular orientation in the GCL, as seen in Fig 6(b). Specifically, the activation due to orientation is influenced via differences in the second spatial derivative of the extracellular potential, which manifests in the frequency domain in Eq (5a) as $-k_{y}^{2}{\hat{\phi }}_{\alpha }$. This analysis also shows that modulating the spread and orientation of the electric field by driving multiple, aligned stimulating electrodes can be used to minimize activation of fibers with specific orientations, such as passing axons.

A secondary effect of using multiple electrodes aligned with passing axons is that the ratio of depolarization of perpendicular AISs to parallel AISs increases with the number of electrodes. In the results summarized in Fig 6, the ratio of the maximal depolarization for perpendicular AISs to parallel AISs is 1.9, 2.1, and 2.5 for one-, two-, and four-electrode configurations, respectively. The cause of the increase from one to four electrodes is likely that, by aligning electrodes with passing axons, the activity of similarly oriented AISs in the GCL is also reduced, while having little effect on the depolarization of perpendicular AISs. This effect is far less pronounced for parallel fibers in the GCL when compared to the NFL due to the natural spread of current at greater retinal depths; the artificial spread of current introduced by using multiple electrodes is less pronounced when compared to the natural longitudinal spread caused by the geometry of the NFL. In contrast, the anisotropic spread introduced by the NFL, shown in Fig 5, has little effect on superficial AOPs as they are close to the retinal surface where current spread is still predominantly isotropic and so we must rely on electrode configuration to control the profile of extracellular potential.

A related phenomenon is highlighted in Fig 8(c), which shows that, given a certain level of GCL activation, there is an increase in the spread of activation as electrodes are moved further from the retinal surface; however, this increase is not seen for four electrodes. The reason for this is that, due to the wider distribution of current at the electrode array when recruiting four electrodes, the increase in spread due to greater electrode-retina distance is marginal. Another key feature of the system is that the region in which the largest spread occurs is in the NFL, in the direction of passing axons. Therefore, an increase in the distance of vitreous fluid through which current flows has a less pronounced effect on total spread in that direction. An increase in the spread of activation in the direction perpendicular to passing axons can be observed as electrodes are moved away from the retina; however, spread is always more pronounced in the direction of passing fibers.

Although these results are based on simulations of cylindrical neurites, the developed method for the analysis of arbitrarily rotated fibers can be applied directly to the simulation of unbranched axons with arbitrary morphology, as discussed in S3 Appendix. A preliminary next step will be to validate the current results using ganglion cell axon reconstructions. A key point of interest will be whether the effect is maintained when axonal orientation changes along the length of the simulated fiber. This will depend on the length constants associated with both axon curvature and membrane activation. If the latter is relatively smaller, axon curvature will have little effect and localized fiber orientation will determine the level of activation along the axon.

### Choosing a stimulation strategy

As can be appreciated from Fig 7(a)–7(c), of the electrode configurations that were simulated, preferential stimulation with clinically desirable parameters can only be achieved with four electrodes. Ideally, electrodes should be placed as close as possible to the surface of the retina without causing damage. This reduces the required stimulus current and limits current spread, thereby increasing the achievable device resolution. From Fig 7(c), most of the change in AIS activation with varying electrode height is seen to occur in the first 100 *μ*m, suggesting that the optimal electrode height considering both preferential AIS activation and activation radius is around 100 *μ*m. Beyond this height, little is gained in terms of preferential activation, with reductions in resolution and larger required stimulus currents.

Given the electrode-electrode separation used in this study, for separation distances of less than 50 *μ*m, preferential stimulation is limited due to a lack of lateral summation of currents from adjacent electrodes; activation under each electrode will occur in a similar way to one electrode. This highlights the fact that these results rely on current spread from adjacent electrodes overlapping and summing together. The level of this summation depends on both the distance between electrodes (the *x*-*y* distance that current must spread) and the distance from the electrodes to the retinal surface (the *z* distance over which current can spread). In theory it is expected that, in the limit of infinitesimally small electrodes that are infinitesimally close together, preferential activation could be achieved with electrodes arbitrarily close to the retinal surface. In reality, the optimal electrode-retina separation distance will depend on the geometry of the electrode array and may differ from the results presented here.

The combination of results shown in Figs 7 and 8 provide a starting point for choosing a clinically relevant stimulation strategy. If the height of the electrode array above the retina is fixed at 100 *μ*m and pulse duration is greater than 50 *μ*s, the chosen pulse duration has little influence on activation provided appropriate current magnitudes are delivered. Key remaining considerations are the required current or charge density, level of activation in the GCL, and size of the activated region, which can be determined from Fig 8. It is unclear exactly how either GCL activation level or activation radius in the current model will map to perception by patients with an implanted device. As such, a suitable stimulus charge may need to be determined either experimentally or based on direct feedback from device users. A suitable charge density will depend on the trade-off between GCL activation level and activation radius (Fig 8(c)), and should always be kept below the level required for AOP-related perception and within clinically determined safety limits. A conservative and commonly cited limit based on the theoretical non-gassing limit is 0.35 mC/cm^2^ per phase for platinum electrodes [59]. As an example, for four-electrode stimulation with an array positioned 100 *μ*m above the retina, this upper-bound is 0.18 mC/cm^2^ per phase, as indicated by the transition from solid to dotted lines in Fig 8(a) or 8(b). A valuable implication of using four-electrode stimulation is that it results in an approximately 3x times decrease in the required charge density and hence is consistently below safe charge density limits. Although the total current and total power required is slightly more for four-electrode stimulation when compared to one-electrode stimulation, this increase is well below proportional.

Although Fig 8 shows that by recruiting more stimulating electrodes the induced area activated becomes greater, it should be noted that this will not necessarily reduce perceived resolution. Previously, recipients of epiretinal implants have reported elongated and line-like phosphenes, thought to be caused by stimulation of passing axons in the NFL that originate from distant regions of the GCL [2, 11–19]. Hence, despite an increase in the region of activation in the GCL when using a four-electrode stimulation strategy, the overall resolution is expected to increase due to the elimination of activation of the NFL. Furthermore, phosphene regularity is expected to be greater under the proposed strategy, more readily facilitating the development of more complex stimulus patterns built up from this perceptual subunit.

### Determining membrane thresholds

To the best of our knowledge, although threshold stimulus currents have been reported for the AIS and distal axon of RGCs (as by Fried et al. [23] and Grosberg et al. [10]), there exists no experimental data on the membrane thresholds of RGCs at these locations. In previous modeling studies, the low threshold of the AIS has only been incorporated into active, conductance-based models of RGCs. In these models, the threshold is reduced at the AIS by increasing the sodium channel density by a factor ranging from 2 to 40, generally chosen to reproduce desired physiological responses [15, 19, 60].

As ion channels are not modeled here, the sensitivity of the AIS was adjusted in our passive model by using different membrane potential thresholds. To avoid arbitrarily choosing a reduced threshold for the AIS, membrane threshold levels were determined using an approximate reproduction of the experimental procedure of Fried et al. [23]. This was made possible as the stimulus thresholds reported by Fried et al. [23] were accompanied by measurements of axonal morphology and three-dimensional electrode location. Simulating the experiment using the same modeling framework in which the thresholds were later applied ensured that the chosen values were most representative of the reported experimental data and were relevant to the current model. The ratio of the calculated membrane threshold for the AOP and AIS was approximately 2. Due to the scale of sodium channel densities utilized in previous models, this suggests that our estimation of the range of parameters for which preferential activation is achievable is conservative. This value is dependent on model parameters for extracellular tissue impedance and the assumed location of the RGC in simulations of the Fried et al. experiments. For instance, if the AIS was assumed to be further from the electrode, an lower membrane potential threshold would have been estimated at the AIS. For this reason, the AIS was assumed to be located just below (5 *μ*m) the surface of GCL, yielding the highest, and therefore most conservative, estimate of threshold membrane potential. Furthermore, as demonstrated by the sensitivity analysis presented in the Results, an even more conservative membrane threshold ratio of 1.5 may be used without much loss of effect.

### Experimental validation

Controlled experimental validation of these results requires techniques for the measurement of RGC activation at multiple locations in the retina simultaneously. *In vitro* studies in which the average trajectory of passing axons in the NFL is known will allow for measurements of activation to be taken in the GCL at both the region being targeted by stimulation and at more distant locations that lie under the trajectory of passing axons. Potentially useful methods have also been developed for monitoring GCL activity across the whole retina using calcium imaging [17] or micron-scale electrical imaging of axonal action potential transmission [61, 62]. Recent developments in high-density stimulation and recording, which enable initiation of action potentials in precise locations of a neuron and imaging of their propagation, may also serve as valuable, non-clinical platforms for the validation of a strategy such as this [63, 64]. A challenge with quantitatively validating the result in this paper is that the small distance between the electrode array and the surface of the retina must be very tightly controlled.

Due to the dependence of these results on the anisotropy of the NFL, it is expected that varying the thickness of the NFL will have a marked effect. The chosen NFL layer thickness is based on an approximation of the human retina, and so these results are relevant only to human retinal stimulation. Rodent models used for research and testing of epiretinal implants have thinner NFLs and so the influence of retinal layer orientation will be less pronounced. Although this in no way confounds the current findings, it suggests that experimental validation would be best carried out in the primate retina. A potential solution for other animal models may be to modify the present model to represent the appropriate animal model so that any observed evidence can be extrapolated to human-like retinal geometries. It is important to note that a large part of the present result derives from electrode configuration, which can be kept consistent across different animal models.

In a real-world implanted system, the assumption that the distance between each electrode and the NFL is the same is unlikely to hold. In addition, the electrode-retina separation distance is likely to change over time due to device settling, immune responses and changes to the implant’s environment caused by vitrectomy. Challenges such as these will require careful modification of the idealized solution presented here, and are likely to require either measurement for each patient of the precise three-dimensional location of the electrode array over time using methods such as OCT or measurement of the retina’s electrical response to stimulation. Procedures such as those used by Grosberg et al. [10] for electrical recording of RGCs following stimulation provide a potential solution. This method enables the classification of responses as either AOP activation or AIS/soma activation. This information could then be used to tune the multi-electrode stimulation strategy over time.

### Optimizing electrode currents

For stimulation strategies that utilize more than two electrodes, it is likely that delivering equal currents to all electrodes does not represent the optimal stimulus for achieving preferential activation with minimal activation radius. It may be possible to more optimally distribute currents across the recruited electrodes in a way that minimizes the activating function along AOPs. As can be seen from Fig 8(d)–8(f), for one- and two-electrode configurations, the profile of activation about the center of the electrode array follows a simple curve with monotonic first derivative. In contrast, with four electrodes, the profile has a more complex shape due to the added degree of freedom. In this case, this extra degree of freedom can be represented by the ratio of the current delivered to the two internal and two external electrodes.

As highlighted by Fig 9, there is a range of electrode/AOP orientations that must be dealt with by a proposed stimulation strategy. We have demonstrated that the approach proposed in this paper is robust to changes in relative electrode array to AOP orientation, and can target off-centered tissue volumes. However, it is again likely that delivering equal currents to each electrode is sub-optimal. In this more general case, optimal electrode currents will also depend on the particular pattern of electrodes that is being used. For instance, the optimal ratio of internal electrode currents to external electrode currents for the case presented in Fig 9(b) will be different than for a set of four electrodes perfectly aligned with the AOP.

With four-electrode stimulation, which can be tuned by a single parameter, optimization could be achieved using a simple brute force search through possible current ratios. However, the model presented here is linear and has an analytic solution in the Fourier domain. This means that a closed-form solution to this optimization problem can be found using least squares or some other linear optimization algorithm. This approach could be applied to the optimization of currents delivered to an arbitrary number of electrodes to minimize activation of the NFL. Optimization of multiple electrode currents to achieve both focal activation of the GCL and minimal activation of the NFL will be the subject of a subsequent study.

## Conclusion

This paper demonstrates that activation of RGCs in the inner retina under epiretinal stimulation depends on both axonal orientation and orientation of the stimulating electric field relative to the orientation of AOPs in the NFL. The developed model allows for an analysis of this dependence by capturing the distinct distributions of fiber orientation of the nerve fiber layer and the ganglion cell layer. A four-electrode stimulation strategy has been proposed that accomplishes preferential activation of the retinal ganglion cell AIS over passing axons in the NFL using clinically suitable stimulus charge densities and electrode configurations. Although concessions must be made with regard to activation radius in the GCL, these are relatively minor, and the proposed strategy is expected to enable higher resolutions and more clearly interpretable percepts by users of epiretinal prostheses.

## Supporting information

S1 Appendix Solution of volume equations. (PDF)

S2 Appendix Admittivity of the nerve fiber layer. (PDF)

S3 Appendix Generalization of neurite equations. (PDF)

S1 Fig Alternative non-ideal electrode array placement. (a)-(b) Membrane potential along neurite axes for axons of passage and axon initial segments, with stimulus current chosen to maximally activate initial segments without activating any passing axons. Colors correspond to those in Fig 6(a), with green parallel to axons of passage and brown perpendicular. Insets describe the geometry of each simulation, indicating target region (red), electrodes used (black), and the orientation of axons of passage (orange). (c)-(d) Ganglion cell layer activation level vs. activation radius for non-ideal and ideal (as in Fig 8(f)) geometries. Transitions from solid to dashed lines represent the transitions from axon initial segment to axon of passage preferential activation. (e)-(f) The spread of ganglion cell layer activation in the *x*-*y* plane. The dashed blue line corresponds to the one-dimensional inset. Colors are mapped according to the color bar in Fig 8. The left-hand panel (a, c, and e) shows stimulation with 6 electrodes, each with equal current. The right-hand panel (b, d, and f) shows stimulation of an off-center region of the GCL by halving the current delivered from the bottom row of electrodes. All simulations used a pulse phase duration of 200 *μ*s and electrode-retina separation of 100 *μ*m.
(TIF)
