# Supplementary material for: Minimizing activation of overlying axons with epiretinal stimulation: The role of fiber orientation and electrode configuration
Source: PLoS One. 2018 Mar 1;13(3):e0193598. doi: 10.1371/journal.pone.0193598 (PMC5833203; doi:10.1371/journal.pone.0193598)
Supplement: S1 Appendix — (PDF) [file pone.0193598.s001.pdf]

S1 Appendix Solution of volume equations. Expanding Eq (2) for each layer gives

$$\mathbf{J}_{\mathrm{V}} = \frac{-1}{4\pi^2}\left(\xi_{\mathrm{V}_x} * \frac{\partial \phi_{\mathrm{V}}}{\partial x}\mathbf{e}_x + \xi_{\mathrm{V}_y} * \frac{\partial \phi_{\mathrm{V}}}{\partial y}\mathbf{e}_y + \xi_{\mathrm{V}_z} * \frac{\partial \phi_{\mathrm{V}}}{\partial z}\mathbf{e}_z\right), \tag{S1.1a}$$

$$\mathbf{J}_{\mathrm{N}} = \frac{-1}{4\pi^2}\left(\xi_{\mathrm{N}_x} * \frac{\partial \phi_{\mathrm{N}}}{\partial x}\mathbf{e}_x + \xi_{\mathrm{N}_y} * \frac{\partial \phi_{\mathrm{N}}}{\partial y}\mathbf{e}_y + \xi_{\mathrm{N}_z} * \frac{\partial \phi_{\mathrm{N}}}{\partial z}\mathbf{e}_z\right), \tag{S1.1b}$$

$$\mathbf{J}_{\mathrm{G}} = \frac{-1}{4\pi^2}\left(\xi_{\mathrm{G}_x} * \frac{\partial \phi_{\mathrm{G}}}{\partial x}\mathbf{e}_x + \xi_{\mathrm{G}_y} * \frac{\partial \phi_{\mathrm{G}}}{\partial y}\mathbf{e}_y + \xi_{\mathrm{G}_z} * \frac{\partial \phi_{\mathrm{G}}}{\partial z}\mathbf{e}_z\right), \tag{S1.1c}$$

where $\mathbf{e}_x$, $\mathbf{e}_y$, and $\mathbf{e}_z$ are unit vectors in x, y, and z directions, respectively. Note that each of the dimension-specific admittivity terms in the above expression may have (x, y, z, t) dependence.

By assuming that within each layer tissue admittivity is independent of z, we can reduce the above four dimensional convolutions to three dimensions. Using the x-component of the admittivity in the vitreous layer to illustrate, we have

$$\begin{aligned}\xi_{\mathrm{V}_x} * \frac{\partial \phi_{\mathrm{V}}}{\partial x} &= \sqrt{2\pi} \iiiint\limits_{x'\,y'\,z'\,t'} \bar{\xi}_{\mathrm{V}_x}(x', y', t')\delta(z') \\ &\quad\times \frac{\partial \phi_{\mathrm{V}}(\mathbf{r}-\mathbf{r}', t-t')}{\partial x}\mathrm{d}x'\mathrm{d}y'\mathrm{d}z'\mathrm{d}t' \\ &= \sqrt{2\pi} \iiint\limits_{x'\,y'\,t'} \bar{\xi}_{\mathrm{V}_x}(x', y', t')\frac{\partial \phi_{\mathrm{V}}(\mathbf{r}-\mathbf{r}', t-t')}{\partial x}\mathrm{d}x'\mathrm{d}y'\mathrm{d}t',\end{aligned} \tag{S1.2}$$

where $\bar{\xi}_{\mathrm{V}_x}$ is the x-component of the admittivity in the vitreous layer at a given point in (x, y, t) space, which does not vary with z within a layer. Identical simplifications can be shown for each of the nine convolutions in Eqs (S1.1). Subsequently, this will allow for the removal of these convolutions using a Fourier transform in three dimensions instead of four.

All Fourier domain transformations performed in these analyses are of the following form:

$$F(k_x) = \frac{1}{\sqrt{2\pi}} \int\limits_x f(x)\mathrm{e}^{-jk_x x}\mathrm{d}x, \tag{S1.3a}$$

$$f(x) = \frac{1}{\sqrt{2\pi}} \int\limits_{k_x} F(k_x)\mathrm{e}^{jk_x x}\mathrm{d}k_x, \tag{S1.3b}$$

where k_x and $F(k_x)$ are the Fourier transform pairs of x and $f(x)$, respectively.

Taking the Fourier transform of each of Eqs (1) with respect to x, y, and t gives the following set of equations:

$$\begin{aligned}\hat{\nabla} \cdot \hat{\mathbf{J}}_{\mathrm{V}} &= \sum_{\mathrm{i}}^{\mathrm{M}} \frac{\hat{I}_{\mathrm{i}}(\omega)}{\pi q} \frac{\mathbb{J}_1\big(q\sqrt{k_x^2 + k_y^2}\big)}{\sqrt{k_x^2 + k_y^2}} \\ &\quad\times \delta(z - z_{\mathrm{i}})\mathrm{e}^{-j(k_x x_{\mathrm{i}} + k_y y_{\mathrm{i}})},\end{aligned} \tag{S1.4a}$$

$$\hat{\nabla} \cdot \hat{\mathbf{J}}_{\mathrm{N}} = 0, \tag{S1.4b}$$

$$\hat{\nabla} \cdot \hat{\mathbf{J}}_{\mathrm{G}} = 0, \tag{S1.4c}$$

where the hat symbol $(\hat{\ })$ indicates the Fourier transform of the specified quantity with respect to x, y, and t, with Fourier transform pairs k_x, k_y, and ω, respectively.

$\hat{\nabla} = \begin{bmatrix} jk_x; & jk_y; & \partial/\partial z \end{bmatrix}$ is the Fourier transform of the differential operator and $\mathbb{J}_1(\cdot)$ represents the Bessel function of the first kind of order 1. Similarly, taking the Fourier transform of Equations (S1.1) yields

$$\hat{\mathbf{J}}_{\mathrm{V}} = -jk_x\hat{\xi}_{\mathrm{V}_x}\hat{\phi}_{\mathrm{V}}\mathbf{e}_x - jk_y\hat{\xi}_{\mathrm{V}_y}\hat{\phi}_{\mathrm{V}}\mathbf{e}_y - \hat{\xi}_{\mathrm{V}_z}\frac{\partial\hat{\phi}_{\mathrm{V}}}{\partial z}\mathbf{e}_z, \tag{S1.5a}$$

$$\hat{\mathbf{J}}_{\mathrm{N}} = -jk_x\hat{\xi}_{\mathrm{N}_x}\hat{\phi}_{\mathrm{N}}\mathbf{e}_x - jk_y\hat{\xi}_{\mathrm{N}_y}\hat{\phi}_{\mathrm{N}}\mathbf{e}_y - \hat{\xi}_{\mathrm{N}_z}\frac{\partial\hat{\phi}_{\mathrm{N}}}{\partial z}\mathbf{e}_z, \tag{S1.5b}$$

$$\hat{\mathbf{J}}_{\mathrm{G}} = -jk_x\hat{\xi}_{\mathrm{G}_x}\hat{\phi}_{\mathrm{G}}\mathbf{e}_x - jk_y\hat{\xi}_{\mathrm{G}_y}\hat{\phi}_{\mathrm{G}}\mathbf{e}_y - \hat{\xi}_{\mathrm{G}_z}\frac{\partial\hat{\phi}_{\mathrm{G}}}{\partial z}\mathbf{e}_z. \tag{S1.5c}$$

Substituting Equations (S1.5) into (S1.4), the system may be written as

$$\frac{\partial^2\hat{\phi}_{\mathrm{V}}}{\partial z^2} - \hat{\phi}_{\mathrm{V}}\eta_{\mathrm{V}}^2 = -\sum_{\mathrm{i}}^{\mathrm{M}} m_{\mathrm{i}}\delta(z - z_{\mathrm{i}}), \tag{S1.6a}$$

$$\frac{\partial^2\hat{\phi}_{\mathrm{N}}}{\partial z^2} - \hat{\phi}_{\mathrm{N}}\eta_{\mathrm{N}}^2 = 0, \tag{S1.6b}$$

$$\frac{\partial^2\hat{\phi}_{\mathrm{G}}}{\partial z^2} - \hat{\phi}_{\mathrm{G}}\eta_{\mathrm{G}}^2 = 0, \tag{S1.6c}$$

where

$$m_{\mathrm{i}} = \frac{\hat{I}_{\mathrm{i}}(\omega)}{\xi_{\mathrm{V}_z}\pi q}\frac{\mathbb{J}_1\left(q\sqrt{k_x^2 + k_y^2}\right)}{\sqrt{k_x^2 + k_y^2}}\mathrm{e}^{-j(k_x x_{\mathrm{i}} + k_y y_{\mathrm{i}})},$$

and

$$\eta_\alpha^2 = \frac{k_x^2\hat{\xi}_{\alpha_x} + k_y^2\hat{\xi}_{\alpha_y}}{\hat{\xi}_{\alpha_z}}, \qquad \alpha \in \{\mathrm{V}, \mathrm{N}, \mathrm{G}\}.$$

Solutions to Eqs (S1.6) are of the form shown in Eq (4). By substituting the boundary conditions in Equations (3) into Equations (S1.5) and (4), the following simultaneous equations define the system's constants of integration

$$C_1 = 0, \tag{S1.7a}$$

$$A_1 + A_2 - B_1 - B_2 = -\frac{m_{\mathrm{i}}}{2\eta_{\mathrm{V}}}e^{-\eta_{\mathrm{V}}d_{\mathrm{ER}}}, \tag{S1.7b}$$

$$B_1e^{\eta_{\mathrm{N}}d_{\mathrm{N}}} + B_2e^{-\eta_{\mathrm{N}}d_{\mathrm{N}}} - C_1e^{-\eta_{\mathrm{G}}d_{\mathrm{N}}} = 0, \tag{S1.7c}$$

$$\begin{aligned} &A_1\hat{\xi}_{\mathrm{V}_z}\eta_{\mathrm{V}} - A_2\hat{\xi}_{\mathrm{V}_z}\eta_{\mathrm{V}} - B_1\hat{\xi}_{\mathrm{N}_z}\eta_{\mathrm{N}} + B_2\hat{\xi}_{\mathrm{F}_z}\eta_{\mathrm{F}} \\ &\qquad = \frac{m_{\mathrm{i}}\hat{\xi}_{\mathrm{V}_z}}{2}e^{-\eta_{\mathrm{V}}d_{\mathrm{ER}}}, \end{aligned} \tag{S1.7d}$$

$$\begin{aligned} &B_1\hat{\xi}_{\mathrm{N}_z}\eta_{\mathrm{N}}e^{\eta_{\mathrm{N}}d_{\mathrm{N}}} - B_2\hat{\xi}_{\mathrm{N}_z}\eta_{\mathrm{N}}e^{-\eta_{\mathrm{N}}d_{\mathrm{N}}} \\ &\qquad + C_2\hat{\xi}_{\mathrm{G}_z}\eta_{\mathrm{G}}e^{-\eta_{\mathrm{G}}d_{\mathrm{N}}} = 0, \end{aligned} \tag{S1.7e}$$

$$\begin{aligned} &A_1\hat{\xi}_{\mathrm{V}_z}\eta_{\mathrm{V}}e^{-\eta_{\mathrm{V}}(d_{\mathrm{ER}}+d_{\mathrm{EI}})} - A_2\hat{\xi}_{\mathrm{V}_z}\eta_{\mathrm{V}}e^{\eta_{\mathrm{V}}(d_{\mathrm{ER}}+d_{\mathrm{EI}})} \\ &\qquad = \frac{m_{\mathrm{i}}\hat{\xi}_{\mathrm{V}_z}}{2}e^{-\eta_{\mathrm{V}}d_{\mathrm{EI}}}. \end{aligned} \tag{S1.7f}$$

Given the complexity of the resultant expressions, this set of equations was solved with the symbolic mathematics engine, Mathematica (Wolfram Research, Version 10). Note that the integration constants are obtained separately for each electrode. Owing to the model's linearity, multi-electrode simulations are implemented via the superposition of the electric field generated from several single-electrode simulations. In order to eliminate d_{EI} from the resulting solution, the right limit as d_{EI} goes to zero was also computed with Mathematica.
