# Supplementary material for: Minimizing activation of overlying axons with epiretinal stimulation: The role of fiber orientation and electrode configuration
Source: PLoS One. 2018 Mar 1;13(3):e0193598. doi: 10.1371/journal.pone.0193598 (PMC5833203; doi:10.1371/journal.pone.0193598)
Supplement: S2 Appendix — (PDF) [file pone.0193598.s002.pdf]

S2 Appendix Admittivity of the nerve fiber layer. As shown in Table 1, the insulator, vitreous, and ganglion cell layers are described using scalar conductivities. This is equivalent to these layers having a spatially- and temporally-independent admittivity term that is also isotropic. Like the NFL, the GCL consists of active neural tissue, and hence would be expected to have some capacitive response to stimulation, which would lead to a nonzero permittivity. However, since the GCL is predominantly composed of cell somas, the volume proportion taken up by neural membrane is much lower than that of the NFL, which is predominantly composed of densely packed and thinner fibers. For this reason, the GCL, as for the insulator and vitreous layers, is assumed to have a constant conductance, and is considered to be isotropic:

$$\xi_{\mathrm{I}_x} = \xi_{\mathrm{I}_y} = \xi_{\mathrm{I}_z} = \sigma_{\mathrm{I}}, \tag{S2.1a}$$
$$\xi_{\mathrm{V}_x} = \xi_{\mathrm{V}_y} = \xi_{\mathrm{V}_z} = \sigma_{\mathrm{V}}, \tag{S2.1b}$$
$$\xi_{\mathrm{G}_x} = \xi_{\mathrm{G}_y} = \xi_{\mathrm{G}_z} = \sigma_{\mathrm{G}}. \tag{S2.1c}$$

The cellular composite model provides expressions for both the tissue admittivity kernel for the NFL and for the membrane potential of a neurite given the extracellular potential along its axis (irrespective of which layer the neurite is in) [1].

We assume that in the NFL fibers are oriented in the y-direction. Then, in the time and space domains, the NFL admittivity is given by

$$\begin{aligned} \xi_{\mathrm{N}}(y,t) &= \begin{bmatrix} \xi_{\mathrm{N}_x} & 0 & 0 \\ 0 & \xi_{\mathrm{N}_y} & 0 \\ 0 & 0 & \xi_{\mathrm{N}_z} \end{bmatrix} \\ &= \begin{bmatrix} \xi_{\mathrm{N_T}} & 0 & 0 \\ 0 & \xi_{\mathrm{N_L}} & 0 \\ 0 & 0 & \xi_{\mathrm{N_T}} \end{bmatrix}, \end{aligned} \tag{S2.2}$$

where

$$\xi_{\mathrm{N_T}}(y,t) = \frac{2\pi d}{b\rho_{\mathrm{e}}}\delta(y)\delta(t), \tag{S2.3a}$$

$$\begin{aligned} \xi_{\mathrm{N_L}}(y,t) = &\frac{2\pi\delta(y)\delta(t)}{\rho_{\mathrm{i}}} \\ &- \frac{\sqrt{\pi}H(t)\tau_{\mathrm{m}}^{\frac{3}{2}}}{4\rho_{\mathrm{i}}\lambda_{0_{\mathrm{V}}}t^{\frac{5}{2}}}\left(\frac{2t}{\tau_{\mathrm{m}}} - \frac{y^2}{\lambda_{0_{\mathrm{V}}}^2}\right)e^{-\frac{t}{\tau_{\mathrm{m}}} - \frac{y^2\tau_{\mathrm{m}}}{4\lambda_{0_{\mathrm{V}}}^2 t}}, \end{aligned} \tag{S2.3b}$$

in which $\xi_{\mathrm{N_T}}$ and $\xi_{\mathrm{N_L}}$ are the transverse and longitudinal components of the admittivity kernel, respectively, and H is the Heaviside step function. The remaining terms, d, b, ρ_{e}, ρ_{i}, τ_{m}, and $\lambda_{0_{\mathrm{V}}}$ each represent different physical or electrical properties of the tissue and are defined in Table S2.1. The above expressions reduce to the following by taking the Fourier transform with respect to y and t:

$$\hat{\xi}_{\mathrm{N_T}}(k_y,\omega) = \frac{d}{b\rho_{\mathrm{e}}}, \tag{S2.4a}$$

$$\hat{\xi}_{\mathrm{N_L}}(k_y,\omega) = \frac{1}{\rho_{\mathrm{i}}}\frac{1 + j\omega\tau_{\mathrm{m}} + k_y^2\lambda_{0_{\mathrm{J}}}^2}{1 + j\omega\tau_{\mathrm{m}} + k_y^2\lambda_{0_{\mathrm{V}}}^2}. \tag{S2.4b}$$

Specific layer admittivities and conductivities define the level of anisotropy for each layer as well as unique spatiotemporal dependencies. For the NFL and GCL, the form

of the admittivity represents the assumed distribution of fiber orientations in each layer. The NFL is modeled as a parallel fiber bundle (anisotropic) and fibers in the GCL are modeled as having a uniform distribution of orientations (isotropic). Eqs (S2.3) describe a non-local, non-instantaneous admittivity for the NFL, which is derived from an accurate characterization of the spatiotemporal electrical properties of individual neurites that comprise the tissue [1].

Table S2.1. Model parameters

Parameter	Description	Unit
a	Neurite radius	m
b	Outer cylinder radius	m
d	Width of extracellular sheath, $d = b - a$	m
ρ_i	Intracellular resistivity	Ω m
ρ_e	Extracellular resistivity	Ω m
r_i	Intracellular resistance per unit length, $r_\mathrm{i} = \rho_\mathrm{i}/(\pi a^2)$	Ω/m
r_e	Extracellular resistance per unit length, $r_\mathrm{e} = \rho_\mathrm{e}/(\pi(b^2 - a^2))$	Ω/m
R_m	Membrane unit area resistance	Ω m^2
r_m	Membrane unit length resistance, $r_\mathrm{m} = R_\mathrm{m}/(2\pi a)$	Ω m
C_m	Membrane capacitance per unit area	$\mathrm{F/m^2}$
τ_m	Membrane time constant, $\tau_\mathrm{m} = R_\mathrm{m} C_\mathrm{m}$	s
λ_{0_J}	Static electrotonic length constant for current density boundary conditions, $\lambda_{0_\mathrm{J}} = \sqrt{r_\mathrm{m}/(r_\mathrm{e} + r_\mathrm{i})}$	m
$\lambda_\mathrm{J}(\omega)$	Frequency-dependent electrotonic length constant for current density boundary conditions, $\lambda_\mathrm{J}(\omega) = \lambda_{0_\mathrm{J}}/\sqrt{1 + j\omega\tau_\mathrm{m}}$	m
λ_{0_V}	Static electrotonic length constant for voltage boundary conditions, $\lambda_{0_\mathrm{V}} = \sqrt{r_\mathrm{m}/r_\mathrm{i}}$	m
$\lambda_\mathrm{V}(\omega)$	Frequency-dependent electrotonic length constant for voltage boundary conditions, $\lambda_\mathrm{V}(\omega) = \lambda_{0_\mathrm{V}}/\sqrt{1 + j\omega\tau_\mathrm{m}}$	m
d_N	Nerve fiber layer thickness	m
d_ER	Electrode-retina separation distance	m
d_EI	Electrode-insulator separation, set to zero in final solution	m
q	Radius of disc electrodes	m
$x_\mathrm{i}, y_\mathrm{i}, z_\mathrm{i}$	Location of center of electrode i	m

1. Meffin H, Tahayori B, Sergeev EN, Mareels IMY, Grayden DB, Burkitt AN. Modelling extracellular electrical stimulation: III. Derivation and interpretation of neural tissue equations. J Neural Eng. 2014;11(6):065004. doi:10.1088/1741-2560/11/6/065004.
