# Supplementary material for: Minimizing activation of overlying axons with epiretinal stimulation: The role of fiber orientation and electrode configuration
Source: PLoS One. 2018 Mar 1;13(3):e0193598. doi: 10.1371/journal.pone.0193598 (PMC5833203; doi:10.1371/journal.pone.0193598)
Supplement: S3 Appendix — (PDF) [file pone.0193598.s003.pdf]

S3 Appendix Generalisation of neurite equations. Owing to the high anisotropy of the NFL, fibers with different orientations, whether they are in the NFL or lower layers, will achieve different levels of activation given an applied stimulus. The volume conductor model derived above assumes that membrane potential is calculated for fibers with a single orientation: parallel to the NFL fiber bundle.

The model includes distinct stages for the calculation of extracellular potential (which captures the anisotropy of the NFL) and the calculation of membrane potential. As a result, it is possible to calculate the membrane potential for an unbranched axon with arbitrary morphology if we assume that the orientation of the axon of interest has a negligible effect on the distribution of extracellular potential as a whole. We can first calculate extracellular potential in the region of interest using Eqs (4) and then sample it along the desired axon trajectory. This sampled extracellular potential can then be substituted into Eqs (5) to yield the membrane potential along the axon.

Although this approach achieves a high level of generality, it requires an extra two computationally expensive Fourier transform calculations: one to convert the extracellular potential into the spatial domain from the spatial frequency domain to allow for sampling and another to convert the sampled data back into the frequency domain.

In the case of simple x-y rotations of straight axons, as assumed in the GCL, the required rotation can be performed directly in the Fourier domain, since rotation is preserved under unitary transformations such as the Fourier transform. Extracellular potential is thus calculated using a straightforward modification of Eqs (4) in order to rotate the k_y-axis (and equivalently the y-axis to align with the desired fiber orientation:

$$\begin{aligned}\hat{\phi}_{\mathrm{V}}(k_x', k_y', \omega; z) &= A_1 \mathrm{e}^{-\eta_{\mathrm{V}}(k_x', k_y')z} + A_2 \mathrm{e}^{\eta_{\mathrm{V}}(k_x', k_y')z} \\ &\quad + \sum_{\mathrm{i}}^{\mathrm{N}} \frac{m_{\mathrm{i}}(k_x', k_y', \omega)}{2\eta_{\mathrm{V}}(k_x', k_y')} \mathrm{e}^{-\eta_{\mathrm{V}}(k_x', k_y')|z - z_{\mathrm{i}}|}, \end{aligned} \tag{S3.1a}$$

$$\hat{\phi}_{\mathrm{N}}(k_x', k_y', \omega; z) = B_1 \mathrm{e}^{-\eta_{\mathrm{N}}(k_x', k_y')z} + B_2 \mathrm{e}^{\eta_{\mathrm{N}}(k_x', k_y')z}, \tag{S3.1b}$$

$$\hat{\phi}_{\mathrm{G}}(k_x', k_y', \omega; z) = C_1 \mathrm{e}^{-\eta_{\mathrm{G}}(k_x', k_y')z} + C_2 \mathrm{e}^{\eta_{\mathrm{G}}(k_x', k_y')z}, \tag{S3.1c}$$

where, for a rotation in the x-y plane of θ, we use

$$\begin{bmatrix} k_x' \\ k_y' \end{bmatrix} = \begin{bmatrix} \cos(\theta) & \sin(\theta) \\ -\sin(\theta) & \cos(\theta) \end{bmatrix} \begin{bmatrix} k_x \\ k_y \end{bmatrix}.$$

The corresponding rotated extracellular potential can then be applied directly to Eqs (5).
