# Supplementary figures and images for: Minimizing activation of overlying axons with epiretinal stimulation: The role of fiber orientation and electrode configuration
Source: PLoS One. 2018 Mar 1;13(3):e0193598. doi: 10.1371/journal.pone.0193598 (PMC5833203; doi:10.1371/journal.pone.0193598)

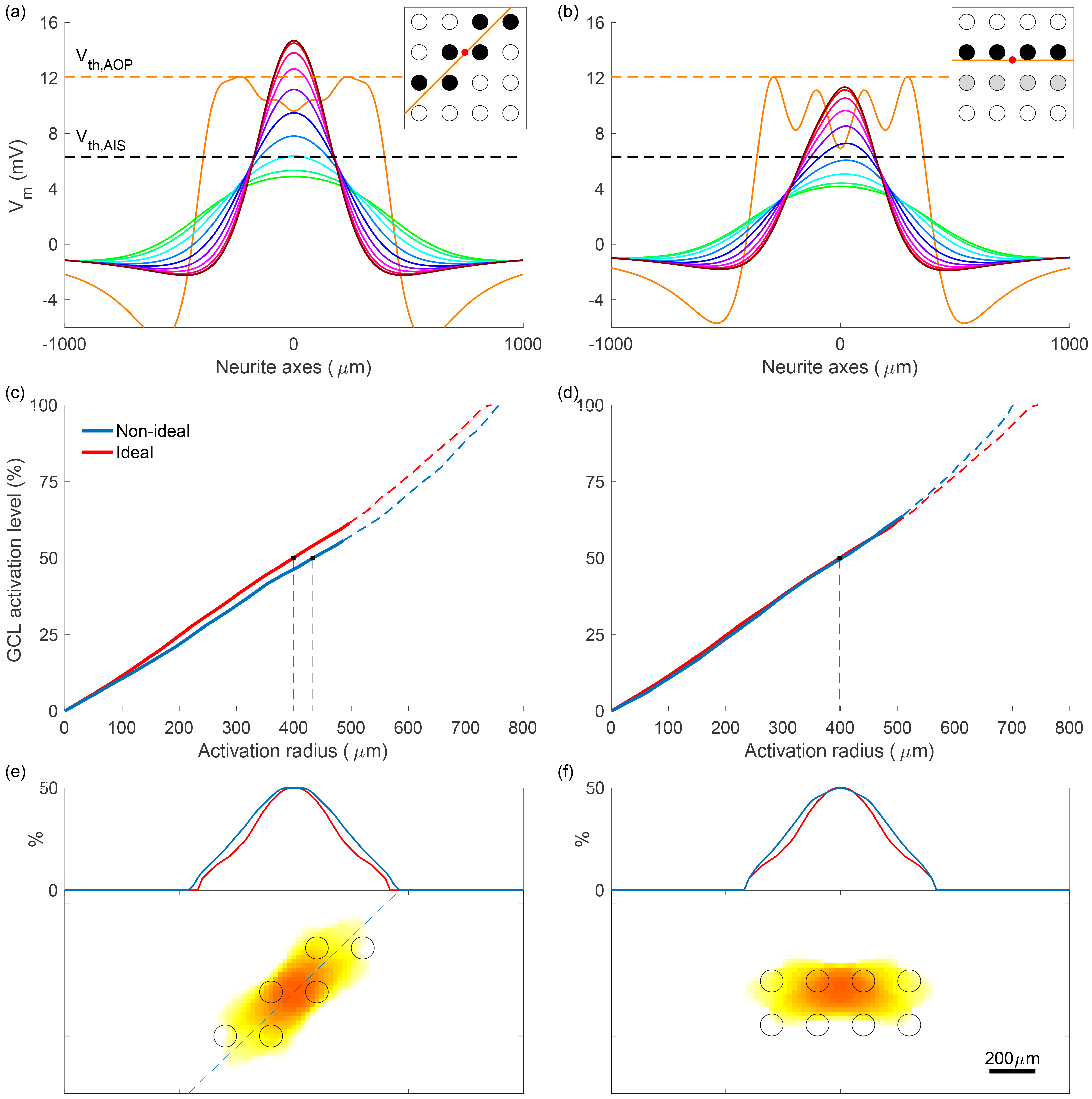

Supplement: S1 Fig — (a)-(b) Membrane potential along neurite axes for axons of passage and axon initial segments, with stimulus current chosen to maximally activate initial segments without activating any passing axons. Colors correspond to those in Fig 6(a), with green parallel to axons of passage and brown perpendicular. Insets describe the geometry of each simulation, indicating target region (red), electrodes used (black), and the orientation of axons of passage (orange). (c)-(d) Ganglion cell layer activation level vs. activation radius for non-ideal and ideal (as in Fig 8(f)) geometries. Transitions from solid to dashed lines represent the transitions from axon initial segment to axon of passage preferential activation. (e)-(f) The spread of ganglion cell layer activation in the x-y plane. The dashed blue line corresponds to the one-dimensional inset. Colors are mapped according to the color bar in Fig 8. The left-hand panel (a, c, and e) shows stimulation with 6 electrodes, each with equal current. The right-hand panel (b, d, and f) shows stimulation of an off-center region of the GCL by halving the current delivered from the bottom row of electrodes. All simulations used a pulse phase duration of 200 μs and electrode-retina separation of 100 μm. (TIF) [file pone.0193598.s004.tif]
